# Characterization of the tandem CWCH2 sequence motif: a hallmark of inter-zinc finger interactions

**DOI:** 10.1186/1471-2148-10-53

**Published:** 2010-02-19

**Authors:** Minoru Hatayama, Jun Aruga

**Affiliations:** 1 Laboratory for Behavioral and Developmental Disorders, RIKEN Brain Science Institute, Wako-shi, Saitama 351-0198, Japan; 2 Saitama University Brain Science Institute, Saitama-shi, Saitama 338-8570, Japan

## Abstract

**Background:**

The C2H2 zinc finger (ZF) domain is widely conserved among eukaryotic proteins. In Zic/Gli/Zap1 C2H2 ZF proteins, the two N-terminal ZFs form a single structural unit by sharing a hydrophobic core. This structural unit defines a new motif comprised of two tryptophan side chains at the center of the hydrophobic core. Because each tryptophan residue is located between the two cysteine residues of the C2H2 motif, we have named this structure the tandem CWCH2 (tCWCH2) motif.

**Results:**

Here, we characterized 587 tCWCH2-containing genes using data derived from public databases. We categorized genes into 11 classes including Zic/Gli/Glis, Arid2/Rsc9, PacC, Mizf, Aebp2, Zap1/ZafA, Fungl, Zfp106, Twincl, Clr1, and Fungl-4ZF, based on sequence similarity, domain organization, and functional similarities. tCWCH2 motifs are mostly found in organisms belonging to the Opisthokonta (metazoa, fungi, and choanoflagellates) and Amoebozoa (amoeba, *Dictyostelium discoideum*). By comparison, the C2H2 ZF motif is distributed widely among the eukaryotes. The structure and organization of the tCWCH2 motif, its phylogenetic distribution, and molecular phylogenetic analysis suggest that prototypical tCWCH2 genes existed in the Opisthokonta ancestor. Within-group or between-group comparisons of the tCWCH2 amino acid sequence identified three additional sequence features (site-specific amino acid frequencies, longer linker sequence between two C2H2 ZFs, and frequent extra-sequences within C2H2 ZF motifs).

**Conclusion:**

These features suggest that the tCWCH2 motif is a specialized motif involved in inter-zinc finger interactions.

## Background

Zinc finger (ZF) domains (ZFDs) are found in a large number of eukaryotic proteins [1-3]. A single ZF normally forms a globular structure that is stabilized by binding a zinc ion. Many classes of ZFs have been described in public databases (Pfam, http://pfam.sanger.ac.uk/; Prosite, http://au.expasy.org/prosite/; Interpro, http://www.ebi.ac.uk/interpro/; SMART, http://smart.embl-heidelberg.de/), with Cys-Xn-Cys-Xn-His-Xn-His (C2H2) being one of the most common. Most C2H2 ZF proteins contain tandem arrays of the C2H2 motif, which are located at specific intervals to form a functional domain. ZFDs were originally identified as the DNA-binding domain of transcription factor IIIA (TFIIIa) and other transcription factors, but accumulating evidence suggests that ZFDs also bind RNA and proteins [4-6]. Since ZFDs are essential for many biological processes, their structure-function relationships have been well studied. For the DNA-binding ZFs, the structures of ZFD-DNA complexes have been elucidated for GLI [7], TFIIIA [8], zif268 [9], YY1 [10], and WT1 [11]. These findings have enabled the development of synthetic ZF proteins as versatile molecular tools [12]. Despite their important and widespread roles, the structural basis of ZFD-protein interactions is less understood than those of ZFD-DNA interactions.

The identification of the critical structural features of protein-binding ZFDs remains elusive [5] but some clues are available for a group of ZFs that mediate protein-to-protein interactions [4-6]. Previously, we studied the intra-molecular protein-to-protein interaction between two adjacent C2H2 ZFs [13]. The two N-terminal ZFs (ZF1 and ZF2) of human ZIC3, which has a ZFD composed of five ZFs (ZF1-ZF5), form a single structural unit through a common hydrophobic core. This ZF-connecting hydrophobic core is associated with two tryptophan residues, each of which is located between the zinc-binding cysteine residues in ZF1 and ZF2. Mutation of the tryptophan in ZF1 (W255G) perturbs the subcellular localization and function of the protein, and is associated with a pathological and congenital heart malformation [13,14]. The importance of these tryptophan residues is further supported by their conservation among more than 40 Zic proteins identified in a wide range of eumetazoan species [15].

Previous studies have revealed that human GLI1 (PDBID: 2GLI) and yeast Zap1 (PDBID: 1ZW8) also possess ZFs with two tryptophans in the corresponding region and that these tryptophans are located in the hydrophobic core formed by two adjacent ZFs [16]. These data raise the possibility that domains containing the consensus sequence "Cys-X-Trp-Xn-Cys-Xn-His-Xn-His-Xn-Cys-X-Trp-Xn-Cys-Xn-His-Xn-His", which we have named the tandem CWCH2 (tCWCH2) motif, are involved in the interaction between the two adjacent ZFs. Since our knowledge of the tCWCH2 motif is limited to a small group of proteins, the biological significance of the tCWCH2 structure is unclear.

In the present study, we performed a computer-based analysis of the tCWCH2 motif using sequence data derived from public databases. We classified the tCWCH2-containing sequences into gene classes and then determined their conservation status in each protein family and their phylogenic distribution. We also identified sequence features unique to tCWCH2-containing ZFDs. The significance of the tCWCH2 motif is discussed in terms of its possible structural and functional roles.

## Results

### Three-dimensional structure of known tCWCH2 motifs

**Figure 1:**
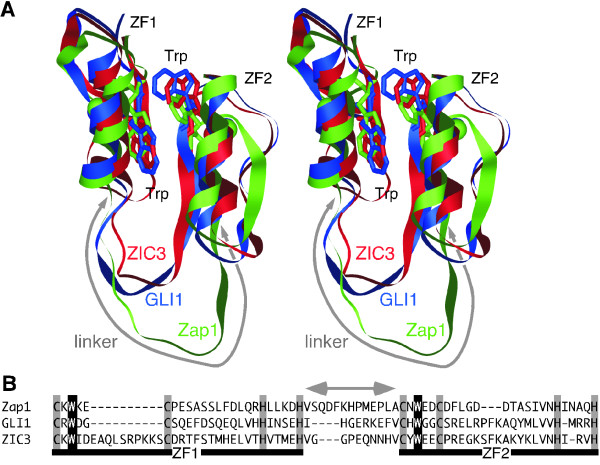
**Structures of tCWCH2 sequence motifs from three different proteins**. (A) Superimposition of the 3D structures of ZIC3, GLI1, and Zap1 tCWCH2 is shown in stereo view. Backbones of the protein structures are indicated by the flat ribbon model. The side chains of two conserved tryptophan residues in tCWCH2 are indicated by the stick model. Red = ZIC3 (PDBID: 2RPC); Blue = GLI1 (PDBID: 2GLI); Green = Zap1 (PDBID: 1ZW8). (B) Amino acid sequence alignment of the tCWCH2 regions. Zinc-chelating cysteine and histidine residues are shown in gray boxes and conserved tryptophan residues are shown in white letters with black boxes. In (A) and (B), the gray lines with arrowheads indicate the sequence between the two CWCH2 motifs (linker sequence).

To investigate the positions of the two key tryptophan residues in different tCWCH2 motifs, we performed a structural alignment to compare the three-dimensional (3D) structures of the tCWCH2 motifs from ZIC3 (PDBID: 2RPC), GLI1 (PDBID: 2GLI) and Zap1 (PDBID: 1ZW8) ZFDs (Figure 1A) with that of two C2H2 ZFs. Our data show that all of these C2H2 ZF motifs form globular structures composed of two anti-parallel β sheets and an α helix (ββα). The two tryptophan residues between the two zinc-chelating cysteine residues (Figure 1B) were localized onto the anti-parallel β sheet where they were juxtaposed to each other. The relative positions of the two tryptophans were similar among the three tCWCH2 structures, as was each zinc-chelating residue. The tryptophan side chains were close to the zinc-chelating histidine in the same ZF, and the hydrophobic residues in the same and the opposite ZF (within 5Å, Additional file 1). This result suggested that the tryptophans play a role in both the stabilization of their own ZFs and the formation of a hydrophobic core between the two adjacent ZFs. The conserved positioning of the tCWCH2 motif-forming residue in evolutionarily distant sources led us to investigate its phylogenetic distribution and structural features.

### Database search for tCWCH2-containing sequences

**Figure 2:**
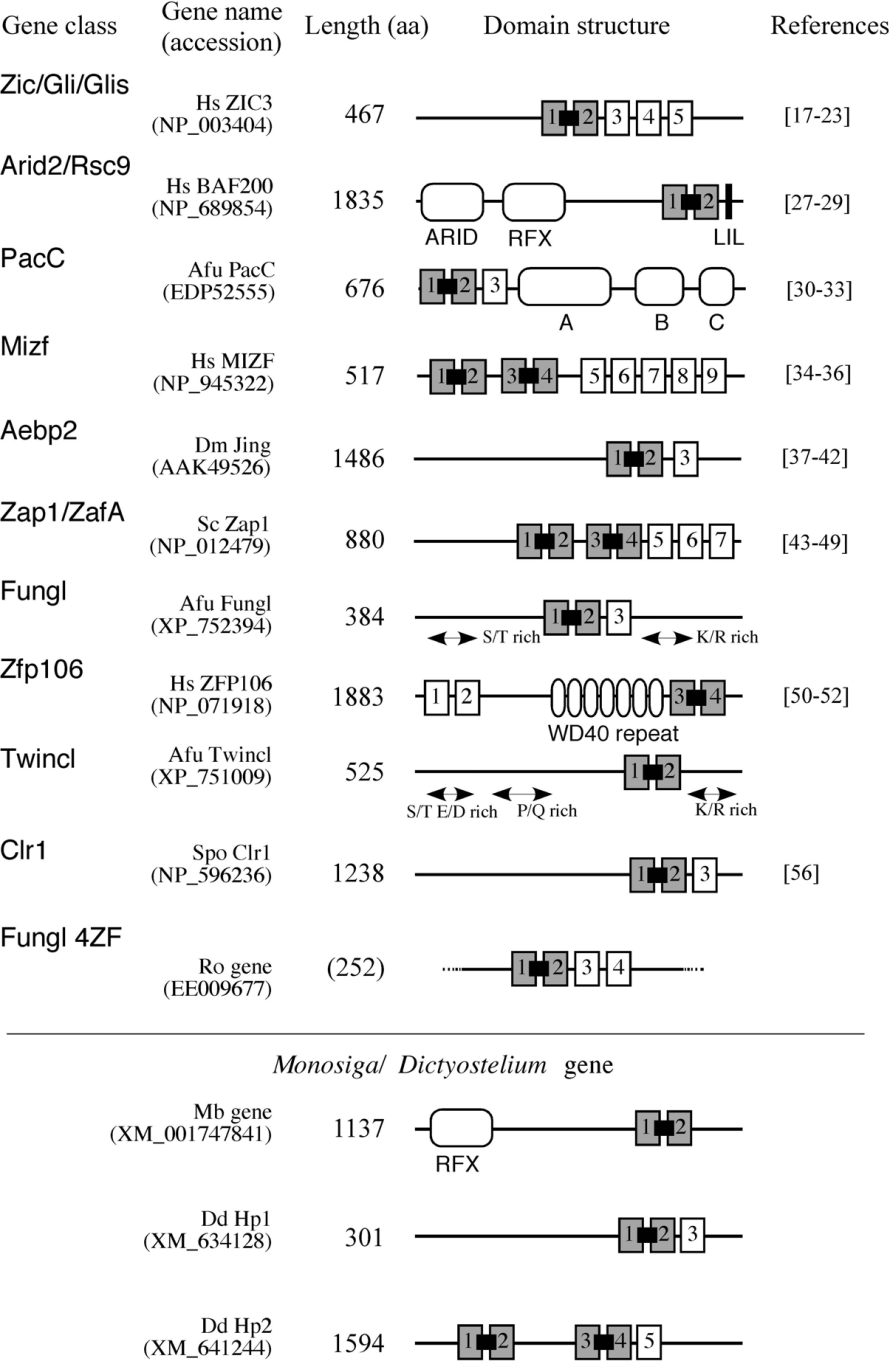
**Domain structure and function of tCWCH2-containing proteins**. The 11 gene classes are listed in descending order of the number of representative genes in each class. A *Monosiga *gene and two *Dictyostelium *genes, which do not belong to these classes, are indicated. Gene name is indicated at the left of each row (open box = C2H2 ZF; gray box = CWCH2; gray boxes linked with thick lines = tCWCH2; open box with curved ends = other domains). Representative genes and their amino acid length are indicated on the left and references are shown on the right. Hs, *Homo sapiens*; Dm, *Drosophila melanogaster*; Sc, *Saccharomyces cerevisiae*; Afu, *Aspergillus fumigatus*; Spo, *Schizosaccharomyces pombe*; Ro, *Rhizopus oryzae*; Mb, *Monosiga brevicollis*; Dd, *Dictyostelium discoideum*.

We performed a comprehensive search of tCWCH2 motif-containing genes in current databases. We performed an initial search of the non-redundant NCBI protein database with PHI-BLAST using the tCWCH2 pattern deduced from the ZIC comparison and human ZIC3 (NP_003404) as the query sequence. This search yielded a set of 709 amino acid sequences that were tentatively classified into 11 gene classes according to their annotations and the pilot phylogenetic tree analyses (see Methods, Figure 2). For the fine analysis of the tCWCH2-containing sequences, we performed TBLASTN and PBLAST searches of the non-redundant sequence collection of the NCBI database using the representative sequences of the initial 11 gene classes and 3 specific genes (two *Dictyostelium *genes and one *Monosiga *gene) as the key sequences. In order to examine the distribution of the tCWCH2 sequence motif in a phylogenetic tree of eukaryotes, TBLASTN searches were also performed on a whole-genome shotgun sequence database of 24 organisms. The sequences obtained were manually checked to remove any redundant sequences. The final tCWCH2 collection contained 587 sequences that represented the non-redundant tCWCH2-containing sequences from a wide range of organisms (Additional file 2). Because the Mizf and Zap1/ZafA family genes and one *Dictyostelium *gene contain two independent tCWCH2 motifs, our collection included a total of 637 independent sequence regions for tCWCH2.

At this point, we re-evaluated the initial sequence pattern for PHI-BLAST because it was based solely on the sequence compilation of the Zic family genes. The initial PHI-BLAST pattern recovered 90% (572/637) of the collected sequences (Additional file 3). Among the 65 non-matching sequences, 20 sequences showed deviation in the C, W, and H residues of the tCWCH2 motif and 50 sequences showed derangement of the sequence length between the two CWCH2 motifs. This mismatch appeared to be distributed randomly and suggested that optimization of the PHI-BLAST pattern was unlikely to be informative. Therefore, we optimized the lengths of the intervening sequences. PHI-BLAST searches using the original pattern "xCxWx(2,35)Cx(5,15)Hx(2,5)Hx(5,25)CxWx(1,3)Cx(5,17)Hx(2,5)Hx" with a tCWCH2 consensus sequence (see Methods) yielded 911 sequences, of which 459 sequences were included in our non-redundant tCWCH2 collection. This number corresponded to 83% of the tCWCH2 sequences in the NCBI protein database (total = 551). A looser pattern, "xCxWx(1,35)Cx(5,15)Hx(2,5)Hx**(5,42)**CxWx**(1,6)**Cx**(5,31)**Hx(2,5)Hx" yielded 968 sequences that contains 469 tCWCH2 sequences (85% of the total) (Additional file 3). The sequences identified by the looser pattern included Zfp407 sequences that contain a pair of CWCH2 ZFs separated by another two C2H2 ZFs. These data suggest that decreasing the stringency of the search (increasing the intervening arbitrary residue number) impairs the fidelity of the search. Therefore, we consider that the original PHI-BLAST pattern is more useful for the tCWCH2 survey.

### Classification and domain structure of tCWCH2 sequence-containing proteins

We classified the non-redundant tCWCH2-containing sequences based on the available flanking amino acid sequences of the tCWCH2 domain (Figure 2). Each gene contained one or two tCWCH2 motifs among a total of 2-9 C2H2 ZF motifs. Proteins containing only one tCWCH2 domain suggest that a single tCWCH2 constitutes the minimal ZFD component. In the ZFDs composed of both tCWCH2 and non-tCWCH2 C2H2, tCWCH2s were always placed at the N-terminal end of the clustered ZFDs.

The 587 sequences included one fungus/metazoan common gene family (Arid2/Rsc9), four metazoan gene families (Zic/Gli/Glis, Mizf, Aebp2, Zfp106), three fungus gene families (PacC, Zap1/ZafA, Clr1), two social amoeba genes (named here as DdHp), and one *Monosiga *gene. In addition, we identified three novel fungal gene groups (*Tandem-CWCH2-protein C-terminal*, *Fungus-Gli-like *and *Fungus-Gli-like-4ZF*). We have summarized the structural features of each tCWCH2 gene class together with major gene functions below and in Figure 2.

### Function, structure, and classification of the gene classes containing tCWCH2

#### Zic/Gli/Glis

The proteins encoded by these three gene classes mediate various processes in animal development [17-23]. Their ZFD is commonly composed of five C2H2 ZFs, and can bind DNA [7,24-26].

#### Arid2/Rsc9

Arid2/Rsc9 genes are known to encode components of an ATP-dependent chromatin remodeling complex [27-29]. The ARID (**A**T-**r**ich **i**nteraction **d**omain) and tCWCH2 domains are conserved in Arid2 and Rsc9 (Additional file 4). Another conserved sequence motif was found in the C-terminal flanking region of the tCWCH2 motif. This sequence motif is summarized as "IxL (S/T) AxL (I/V) L (K/R) N (I/L) × (K/R)" and we named this motif the LIL domain. PHI-BLAST searches for LIL domains suggested that this domain was distributed only in the Arid2/Rsc9 proteins (data not shown).

#### PacC

PacC mediates gene expression regulation by ambient pH in filamentous fungi and yeasts [30,31]. It contains an N-terminal DNA binding domain with three ZFs. Other ZF domains, named A, B, and C, are involved in responses to pH change [32,33].

#### Mizf

Mizf was originally reported as a seven ZF-containing protein [34-36]. We identified two additional C2H2 ZFs in the gene sequence encoding this protein (ZF2 and ZF8 in Additional file 5). The two tCWCH2 motifs in the first four ZFs (ZF1-ZF4) were separated by longer intervening sequences (>35 aa) than most ZFs.

#### Aebp2

Mammalian Aebp2 is a DNA binding transcription factor [37]. Its *Drosophila *homolog, jing, is required for cellular differentiation [38-42].

#### Zap1/ZafA

The proteins encoded by Zap1/ZafA should be grouped together because of the similarity in structure of their ZF domains (Additional file 6) and similarities in their functional roles in zinc homeostasis [43-49]. Our alignments of Zap1/ZafA sequences from seven fungal species (*Ustilago maydis*, *Cryptococcus neoformans*, *Aspergillus fumigatus*, *Magnaporthe grisea, Candida albicans, Saccharomyces cerevisiae *and *Yarrowia lipolytica*) reveal significant similarity in their ZFDs (Additional file 6). The ZFDs contain a maximum of eight C2H2 ZF motifs in which the two N-terminal ZF pairs (ZF1-ZF2, ZF3-ZF4) can form tCWCH2 motifs. ZF1-2 and ZF3-4 are separated by longer intervening sequences than most ZFs. Our alignments showed that conservation of ZF1, ZF2, and ZF8 was incomplete.

#### Fungus-Gli-like (Fungl)

The amino acid sequences encoded by this group of genes have been known as "fungus Gli like". Here, we have named these sequences Fungl (**Fun**gus-**G**li-**l**ike) (Additional file 7, data not shown).

#### Zfp106

Zfp106 is a metazoan ZF protein of unknown function [50,51]. It contains two pairs of ZFs at each end. WD40 repeats (IPR001680, Zfp106) are known to mediate protein-protein interactions [52-55].

#### Tandem-CWCH2-protein-C-terminal (Twincl)

The sequences are composed of 468 to 1458 amino acid residues (data not shown). On the basis of these structural features (Figure 2, Additional file 8), we named this gene family Twincl (**T**andem C**W**CH2 prote**in C**-termina**l**).

#### Clr1

The ZFs among members of the Clr1 family [56] were highly divergent and other sequences were not conserved (Additional file 9, data not shown). Therefore, we omitted this gene family from the consensus sequence and phylogenetic tree analyses.

#### Fungus-Gli-like-4ZF (Fungl-4ZF)

This gene group includes annotations from *Rhizopus oryzae *and *Batrachochytrium dendrobatidis *representing the fungal phyla Mucoromycotina and Chytridiomycota, respectively. The proteins encoded by the Fungl-4ZF gene family contain four ZFs that are weakly similar to those in Gli and Fungl (Additional file 7). Based on the ZF motif organization, we tentatively grouped them separately from Fungl. However, based on their sequence similarity and phylogenetic classification it is possible that the Fungl-4ZF and Fungl gene groups are derived from a common ancestral gene [57,58].

#### Dictyostelium discoideum *tCWCH2*

The social amoeba *Dictyostelium discoideum *belongs to the Amoebozoa supergroup. There are two tCWCH2-containing genes in this gene class, DdHp1 (***D****ictyostelium ****d****iscoideum ***h**ypothetical **p**rotein) and DdHp2. The ZFD alignment of these genes is shown in Additional file 10.

#### Monosiga brevicollis *tCWCH2*

Since *Monosiga brevicollis *is the only species among the choanoflagellates in which the genome has been fully sequenced, the presence of one tCWCH2 sequence (Additional file 10) may be more functionally meaningful than the singleton sequences in metazoa and fungi. The existence of an RFX domain (position 376-423) raised the possibility that the tCWCH2 sequence in *Monosiga brevicollis *and Arid2/Rsc9 is derived from a common ancestral gene.

#### Unclassified sequences

Six sequences were not classified into the above 13 categories (Additional file 2). Four out of the six unclassified sequences contained weak similarities to PacC, Fungl, Fungl-4ZF, and Mizf. The remaining two sequences from fungi did not show any significant homology to the other sequences.

#### Isolated tCWCH2 in metazoan gene families

We found rare occurrences of the tCWCH2 motif in some metazoan gene families, including three tCWCH2-containing sequences in the Sp8 family (n = 37), one in the TRP2 family (n = 8), one in the Krüppel-like factor 5 family (n = 14), and one in the pbrm-1 family (n = 21) (Additional file 2). We did not include these genes in the following analysis because their significance was not clear.

#### tCWCH2 sequences in plant databases

Two plant tCWCH2-containing sequences were found. One sequence (XP_001771543) was the REF6 homolog of a moss (Bryophyta, *Physcomitrella patens*) (Additional file 10). Although REF6 is conserved in many plants, the CWCH2 was not found in other plant species including *Arabidopsis thaliana*, *Oryza sativa*, and *Vitis vinifera *(Additional file 10). The other sequence, AK110182, was from *Oryza sativa *but is highly homologous to PacC. This sequence was otherwise only detected in fungi and therefore might represent contamination of *Oryza sativa *with fungal material.

In the course of the classification procedure, we extracted the sequences belonging to each gene family irrespective of the presence of tCWCH2 motifs identified by BLAST search using representative entire amino acid sequences. Within this group, we examined the extent of conservation of the cysteine, tryptophan, and histidine residues contributing to the tCWCH2 motifs. The analysis indicated that the tryptophans in CWCH2 motif-constituting residues were generally conserved (87%-100%) (Table 1).

**Table 1:** Conservation of the tryptophan residues for the tCWCH2 motif in each gene family.

Gene	n	%
Zic/Gli/Glis	282	99.3
Arid2/Rsc9	61	96.7
PacC	48	100.0
Mizf_ZF12_	40	92.5
Mizf_ZF34_	40	100.0
Aebp2	39	100.0
Zap1/ZafA_ZF12_	10	100.0
Zap1/ZafA_ZF34_	33	97.0
Fungl	32	100.0
Zfp106	16	87.5
Twincl	13	100.0
Clr1	4	100.0
Fungus Gli like 4ZF	2	100.0
*Dictyostelium *gene	3	100.0
*Monosiga *gene	1	100.0

total	624	98.2

^a^The percentages indicate conservation of the tryptophan residues in each gene class. In Mizf and Zap1/ZafA, there are two independent tCWCH2 motifs, for which ZF12 (ZF1-ZF2) indicates the N-terminally located one and ZF34 (ZF3-ZF4) indicates the C-terminally located one.

### Similarity among tCWCH2 gene classes

**Figure 3:**
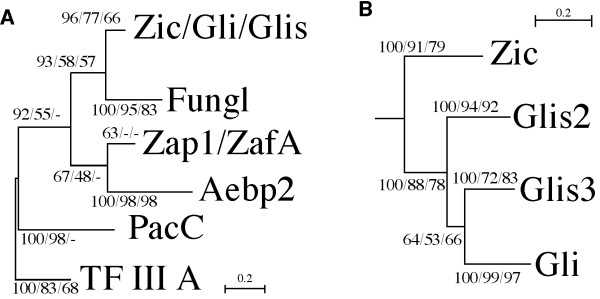
**Phylogenic tree of tCWCH2**. (A) Tree indicating similarities among the tCWCH2 gene classes. Statistical analysis was performed using three ZFs (tCWCH2 and a ZF in the C-terminal flanking region). (B) Sub-tree of Zic/Gli/Glis class gene. The alignment for this analysis is shown in Additional file 11. BI, NL, and NJ analyses were carried out to construct the molecular phylogenetic trees (Additional files 12, 13 and 14). The tree pattern is based on the BI tree. Scores for each branch indicate the statistical support values obtained in each phylogenetic tree construction method {BI (postprobability)/ML (bootstrap value)/NJ (bootstrap value)}. The absence of scores (-) indicates the branches that were not supported by the corresponding methods.

We performed molecular phylogenetic analyses to identify similarities among the tCWCH2 gene classes. For this purpose, we selected flanking ZFs containing tCWCH2 (tCWCH2+1ZF) because the tCWCH2 itself yielded very little information on sequence similarities, presumably because it is short. In addition, genes widely distributed among fungi or metazoa were used for this analysis because our preliminary analysis suggested that genes with a narrow phylogenetic distribution (Twincl, Clr1, Fungl-4ZF, *Dictyostelium *tCWCH2) had strongly divergent sequences that may confound the phylogenetic tree analyses [59]. Using the tCWCH2+1ZF (3ZFs) amino acid sequences, we constructed phylogenetic trees by the Bayesian inference (BI), maximal likelihood (ML), and neighbor joining (NJ) methods. The 89 (80 tCWCH2 + 9 TFIIIA) sequences, representing a wide phylogenic range, were subjected to this analysis using the DNA-binding classical C2H2 ZFs of TFIIIA as outgroup sequences (Figure 3; sequence alignment in Additional file 11; detailed tree in BI, ML, NJ in Additional files 12, 13 and 14).

Each class of tCWCH2-containing genes was strapped with high bootstrap values in the BI, ML, and NJ methods, supporting the validity of the above classification. Phylogenetic tree analyses revealed the relationships among the tCWCH2 gene families and revealed a novel relationship among the metazoan Zic, Gli, and Glis gene families. These three gene families were grouped together, whereas Gli and Glis formed a sub-group in the branch of the Zic/Gli/Glis group. It is known that vertebrate Zic, Gli, and Glis are composed of six, three, and three paralogs, respectively. Interestingly, Glis2 and Glis3 were separately grouped with their insect homologs (DmGlis2 and DmGlis3 respectively) and sea anemone *Nematostella *homologs (NveGlis2 and NveGlis3 respectively; Additional files 12, 13 and 14), indicating that the two paralogs existed in the common ancestors of cnidarians and bilaterians. On the other hand, vertebrate Zic and Gli paralogs were not grouped with the invertebrate homologs (Additional files 12, 13 and 14), suggesting that vertebrate Zic and Gli paralogs were generated in vertebrate ancestors.

Fungl was determined to have sequence similarity with the Zic/Gli/Glis group. The Fungl group branch was always located closest to the Zic/Gli/Glis group in the BI, ML, and NJ trees, but statistical support was weak (BI, 93%). The other gene groups with more than two classes of tCWCH2 motif were not strongly supported (BI, <70%). However, Zap1/ZafA class and Aebp2 class proteins were grouped together by the BI and ML methods, and the PacC family was placed in the basal root of the BI and ML trees beside the other tCWCH2+ZF1 sequences from metazoa and fungi.

### Distribution of the tCWCH2 sequences in the phylogenetic tree

Based on the above analysis, we investigated the phylogeny of the tCWCH2 motif (Figure 3). In global terms, tCWCH2 sequences were detected in all of the organisms we examined in the Opisthokonta supergroup, including metazoa, fungi, choanoflagellate (*Monosiga brevicollis*) and microsporidia (*Encephalitozoon cuniculi*). In the Amoebozoa supergroup, we found two tCWCH2-containing genes in the social amoeba *Dictyostelium discoideum*, but not in *Entamoeba histolytica*. Apart from Opisthokonta and Amoebozoa, two sequences were found in the Plantae databases, but their meaning is not clear. Thus, it appears that the distribution of the tCWCH2 sequence motif is essentially limited to the Uniconta (a collective term for the Opisthokonta and Amoebozoa supergroups) in current sequence databases.

**Figure 4:**
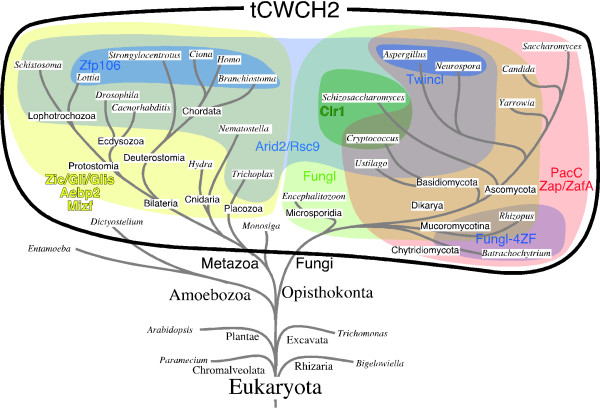
**Distribution of tCWCH2-containing genes in a eukaryotic phylogenetic tree**. Tree pattern (gray curved lines) is based on [78-80]. The distribution of tCWCH2 sequences is indicated by the black curved line. The distribution of each tCWCH2 gene class is indicated by a colored area. The *Monosiga *tCWCH2 sequence may be derived from the common ancestor of Arid2/Rsc9 (See Results).

We examined the distribution of each tCWCH2 motif class in the phylogenetic tree (Figure 4). Arid2/Rsc9 class genes were most widely detected in both fungi and metazoa. On the other hand, tCWCH2 was not detected in the sequences from the order Saccharomycetales (NP_013579, *Saccharomyces cerevisiae*; XP_718578, *Candida albicans*; XP_506030, *Yarrowia lipolytica*). Zic/Gli/Glis, Aebp2, and Mizf were widely distributed in metazoan groups, but Zfp106 was detected only in Bilateralia except Ecdysozoa. PacC, Zap1/ZafA, and Fungl class genes were widely distributed among the fungi groups. Distribution of Twincl class genes was restricted to *Aspergillus *and its close relatives. Clr1 class sequences were found only in *Schizosaccharomyces *and *Cryptococcus*. Fungl-4ZF class genes were recovered only from the basal fungi (*Rhizopus oryzae *and *Batrachochytrium dendrobatidis*).

### Additional sequence features in the tCWCH2 motifs

**Figure 5:**
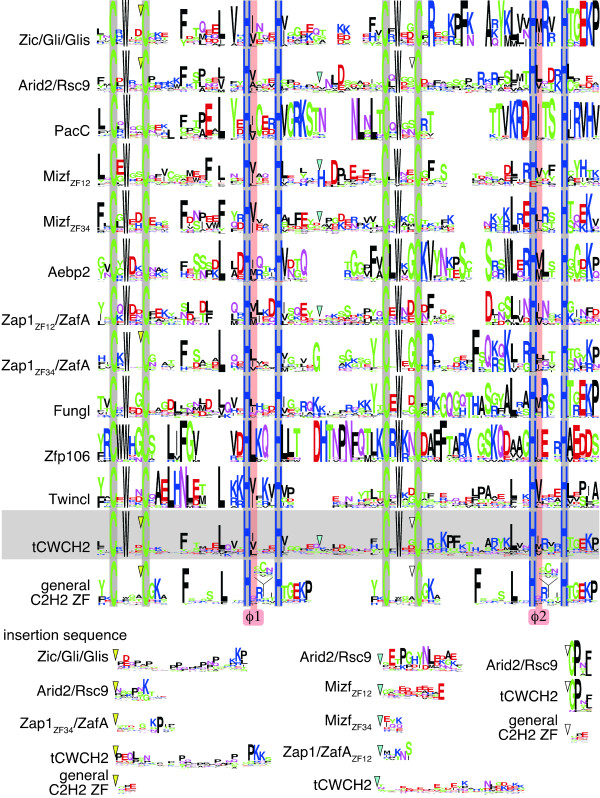
**Sequence conservation among the classes of tCWCH2 sequence motifs**. Amino acid sequences were aligned and consensus sequences were generated (see Methods). Mizf ZF1-2, Mizf ZF3-4, and Zap1/ZafA ZF1-2 and ZF3-4 are indicated separately. The PDOC00028 C2H2 consensus is shown at the bottom as a general C2H2 consensus sequence (indicated by the tandem repeat of the consensus sequence). The short insertion sequences listed below were initially located at the sites indicated by the colored arrowheads in the alignments described above, but were separated to allow more comprehensive analyses. These sequences represent the longer linker sequences and the insertion of extra sequences described in the Results section. To achieve this analysis we omitted the four sequences AAWT01013938 (*Schmidtea mediterranea *Aebp2), CAG05504 (*Tetraodon nigroviridis *Gli), XP_001602003 (*Nasonia vitripennis *Gli), and XP_785526 (*Strongylocentrotus purpuratus *Gli) because these sequences contain exceptionally divergent sequences that disrupt the alignments.

**Figure 6:**
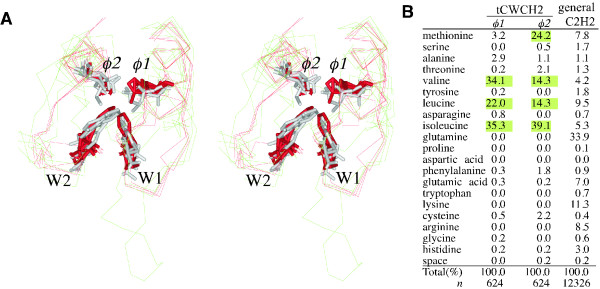
**ϕ1 and ϕ2 positions of tCWCH2**. (A) Stereo view of the tCWCH2 sequence motif. A wire model of the main chains is shown with ZIC3, GLI1, and Zap1 in green and TFIIIA and Zif268 in red. The side chains of tryptophan residues in tCWCH2 and those in the ϕ1, ϕ2 residues are indicated by cylinders. ZIC3, GLI1, and Zap1 are gray cylinders and the others are red cylinders. The corresponding positions (ϕ1, ϕ2) are indicated by red boxes in Figure 5. (B) Frequency of amino acid appearance in ϕ1, ϕ2, and those of general C2H2 (PDOC00028) is shown. Valine, leucine, and isoleucine appear frequently at both positions and methionine frequently appears in ϕ2. This table summarizes all of the tCWCH2 sequences. Highlighted numbers indicate the preferential residues for ϕ1 and ϕ2 in tCWCH2.

We generated tCWCH2 consensus sequences for each gene class as well as for all classes to identify any additional sequence features. The Prosite database PDOC00028 alignment was used for the reference consensus sequence of general C2H2. The extent of conservation is indicated by the size of letters, and the consensus sequences are aligned graphically (Figure 5). Classical C2H2 ZFs are known to have a consensus sequence of "(F/Y)xCx2CxFx7Lx2Hx4H" [1]. The tCWCH2 motifs also contain conserved phenylalanine and leucine residues between the cysteine and histidine residues. Phenylalanine and leucine are hydrophobic residues that generally mediate a hydrophobic interaction within a single ZF (data not shown). These data indicate that the general structure of a single ZF is well conserved in the tCWCH2 motif (Figure 6A) and that there are some tCWCH2-specific structures apart from the conserved tryptophan residues.

Firstly, a position-specific bias to hydrophobic residues was found in the C-terminal region adjacent to the first histidine residue in ZF1 and ZF2 (ϕ1, ϕ2 in Figure 5, 6A). Valine, leucine, and isoleucine were frequently found at ϕ1 position (34.1%, 22.0%, and 35.3%, respectively), and valine, leucine, isoleucine, and methionine were frequently found at ϕ2 position (14.3%, 14.3%, 39.1%, and 24.2%, respectively) in contrast to those in a compilation of general C2H2 sequences (PDOC00028) (valine, 4.2%; leucine, 9.5%; isoleucine, 5.3%; methionine, 7.8%; Figure 6B). The difference in the summed frequency of each position was statistically significant (ϕ1, *P *< 1 × 10^-100^; ϕ2, *P *< 1 × 10^-100 ^in a χ^2 ^test). These positions were close to the tryptophan residues on the opposite side of the ZF as determined in the superimposed 3D structures of Zic, Gli, and Zap1 tCWCH2s (Figure 6A). The biased residue frequency may reflect the spatial restriction of amino acid choices. Since the CWCH2 structure suggests the presence of four hydrophobic residues in a compact space, the residue paired with each tryptophan could not be a residue with a large side chain, such as phenylalanine or tryptophan.

**Figure 7:**
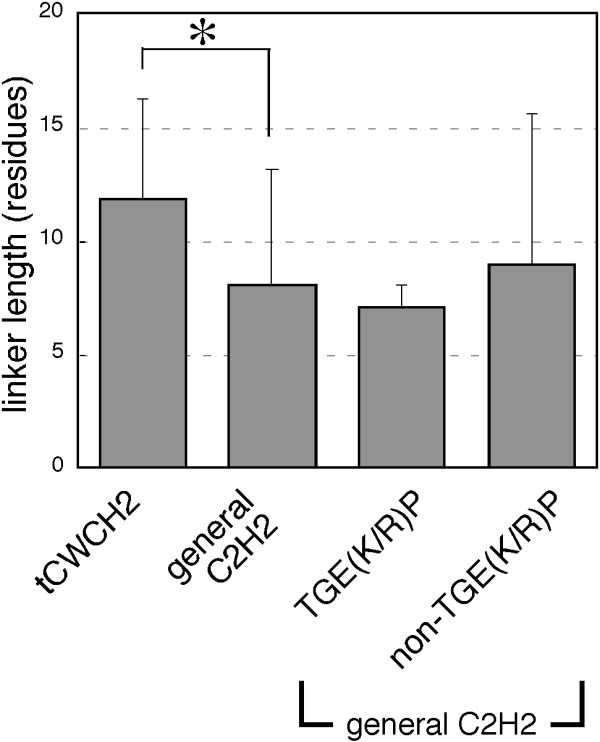
**Comparison of linker length between the tCWCH2 sequence motif and general C2H2 ZF**. The bars indicate the mean lengths of the linker sequence between C2H2 motifs in the indicated groups. We defined the linker length as the number of amino acids between the last histidine of a ZF and the first cysteine of the C-terminally flanking ZF. The PDOC00028 (general C2H2) alignment contained 12326 sequences and we found 10025 linkers in it. The general C2H2 sequences was further divided into two groups based on the presence or absence of "TGE(K/R)P". The sequence numbers of each group: tCWCH2, 614; general C2H2, 10025; "TGE(K/P)", 4640; "non-TGE(K/R)P", 5385). Error bar, standard deviation. *, *P *< 1 × 10^-100 ^in Mann-Whitney U-test.

Secondly, the linker sequence of the two ZFs in tCWCH2 was significantly longer (11.8 ± 4.4 aa, average ± standard deviation) than those of general C2H2 sequences (Prosite PDOC00028, 8.1 ± 5.0 aa) (*P *< 1 × 10^-100^, Mann-Whitney U-test, Figure 7). There were no canonical TGE(K/R)P-like sequences in the tCWCH2 linker sequences, whereas the TGE(K/R)P linker sequence was found in about 65% of the sequences in the PDOC00028 alignment. Accordingly, conservation of the tCWCH2 linker sequences was generally low (Figure 5), and their lengths were more strongly divergent than those of TGE(K/R)P ZFs (*P *= 3 × 10^-7^, F-test). Elongation of the linker was commonly found in the Zap1/ZafA (ZF1-2, 16.2 ± 5.7 aa), Mizf (ZF1-2, 19.2 ± 4.4 aa; ZF3-4, 17.5 ± 2.3 aa) and Arid2/Rsc9 (14.0 ± 6.9 aa) classes.

Thirdly, extra sequences were inserted between the tryptophan and cysteine residues in tCWCH2. This type of insertion was limited to the sequences from the first ZF of Zic/Gli/Glis, Arid2/Rsc9, and Zap1/ZafA and the second ZF of Arid2/Rsc9 classes. The number of amino acid residues between the two cysteines of tCWCH2 varied from four to 34 and was mostly four residues in other classes [15]. This insertion forms an extra looping structure in ZIC3 (PDBID: 2RPC; Figure 1, 6A) [13].

## Discussion

### The tCWCH2 motif is a hallmark of inter-zinc finger interactions

The tCWCH2 motif was originally proposed as an evolutionarily conserved sequence involved in the interaction between ZFs in human ZIC3 [13,15]. The presence of the tCWCH2 motif in the Gli family, Glis family, Zap1, and PacC has been previously reported [13]. Superimposition analysis revealed a tightly conserved structure among ZIC3, GLI, and Zap1, supporting the importance of the tCWCH2 motif in structural terms. Strong conservation of the tCWCH2 tryptophan residues in each gene class suggests that there has been strong selective pressure for them during evolution.

**Figure 8:**
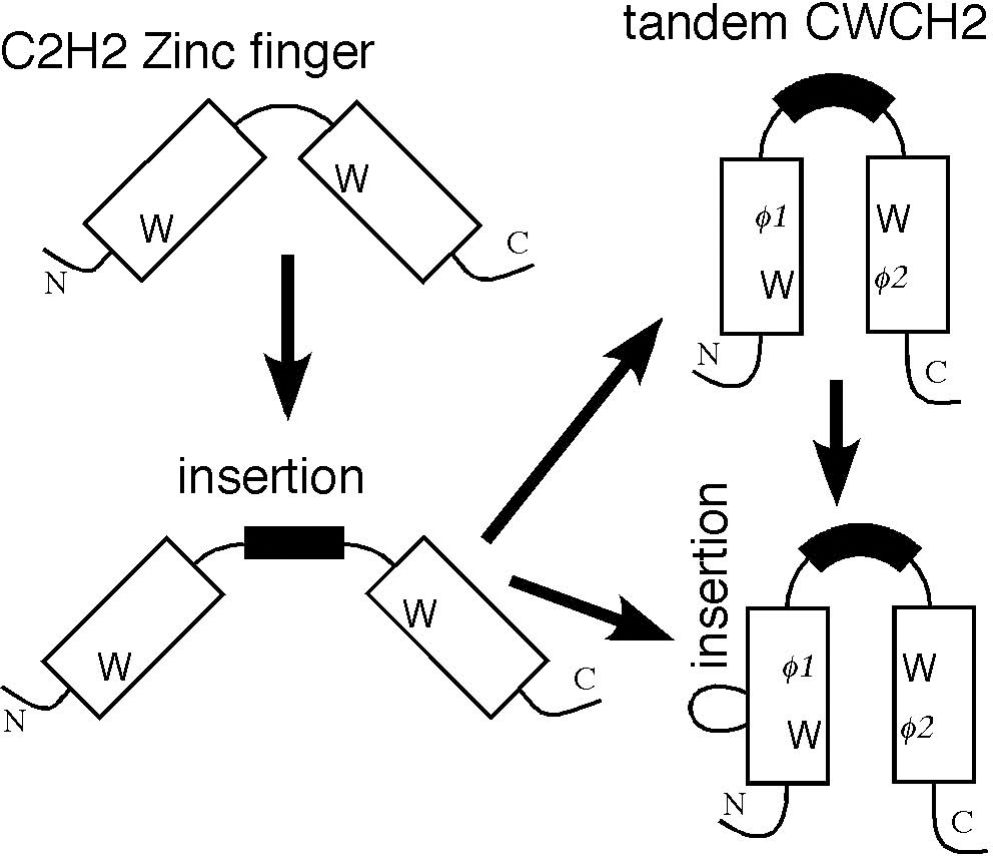
**Evolution of the tCWCH2 domain**. We propose that the tCWCH2 patterns were generated from classical C2H2 sequences concurrently with the acquisition of the additional sequence features.

Our analysis revealed additional structural features of tCWCH2 besides the conserved tryptophans, which may be explained by adaptation of its role in mediating the inter-ZF interaction (Figure 8). Firstly, there is a preference for valine, leucine, or isoleucine in the ϕ1 and ϕ2 position. This can be regarded as an adaptive adjustment for forming hydrophobic cores of the appropriate size. Furthermore, hydrophobic interaction between ϕ1 and ϕ2 is likely because they are closely located within 4Å distance in the 3D structure models of ZIC3, GLI and Zap1 (Additional file 1, data not shown). This also can influence the amino acid preferences in ϕ1 and ϕ2 position. Secondly, elongation of the linker sequences may also reflect a structural adaptation to allow the motif to face the two globular C2H2 units. Linker length and sequence are important for the function of the DNA-binding C2H2 ZF, because they determine the distance of each ZF and the linker contacts to the DNA base [60-62]. However, optimization of the canonical linker length for DNA-binding may not be enough to yield opposed positioning of ZF units. The bending between the two ZFs may be larger than is dictated by the DNA binding state of the ZFs as revealed in the GLI-DNA complex structure [7]. Longer linker size seems to be a general feature of tCWCH2, but conservation of the linker sequences within each class varies notably. Linker conservation in PacC is particularly high, despite the widespread distribution of this gene in fungi, thus suggesting that the linker in PacC is under evolutionary constraint.

Another structural feature, the addition of extra sequences, is plausible if we assume the reduction of structural constraint as a consequence of specialization in the inter-ZF interaction. For example, the conservation of tCWCH2-forming ZF1 is lower than that of ZF2-5 in Zic proteins [15]. In the case of the GLI-DNA complex, the tCWCH2-forming ZF1 does not directly participate in DNA binding [12], and the sequence conservation of Gli family ZF1 is also lower than in ZF2-5 (Figure 5, unpublished observation). The extra sequences in the Zic/Gli/Glis, Zap1/ZafA (ZF3-4), and Arid/Rsc9 classes show poor conservation (Figure 5).

It is unclear if tCWCH2 is the only structure required for inter-ZF interactions. The C2H2 ZFD is capable of achieving protein-to-protein interactions in many cases [6]. Interestingly, in a previous study to design synthetic ZFs utilizing *in vitro*-evolving protein-to-protein interactions [16], it was found that the hydrophobic residues in zif268 that interacted between the artificial proteins and the zif268 C2H2 ZF were similar to those involved in the GLI inter-ZF interaction. Superimposition revealed that the position corresponding to the tryptophan in tCWCH2 is occupied by a valine, suggesting that either intramolecular or intermolecular protein-to-protein interactions, mediated by hydrophobic residues, are generally utilized for C2H2 ZFs. However, the strong conservation of the tCWCH2 and accompanying structural features suggest that tCWCH2 is a highly specialized C2H2 ZF for inter-ZF interactions between two adjacent ZFs.

### Evolution of the tCWCH2 motif

We hypothesize that the original tCWCH2 motifs were generated from classical C2H2 ZFs (Figure 8) that occur widely in eukaryotic organisms, including Plantae, Excavata, Chromalveolata, and Rhizaria. tCWCH2 motifs were found in the vicinity of C2H2 ZF in several tCWCH2 gene classes. It is proposed that the tCWCH2 sequence appeared in the common ancestor of Opisthokonta or Unikonta (Opisthokonta/Amoebozoa). The acquisition of the tryptophans and additional structural features of tCWCH2 may have occurred concurrently in the course of evolution. Whether the evolution of tCWCH2 occurred once or multiple times during Opisthokonta/Amoebozoa evolution remains unclear. However, its short sequence and some isolated distribution in the plant supergroup and metazoan gene families may favor the multiple-origins hypothesis. Although the lineage relationship among the tCWCH2 classes remains unclear, we can presume the presence of the following tCWCH2 ancestral genes: an Arid2/Rsc9 common ancestor, which may have existed before the diversification of metazoa and fungi; a Zic/Gli/Glis common ancestor, an Aebp2 ancestor, and a Mizf ancestor in the early metazoa; a Fungl ancestor, a PacC ancestor, a Fungl-4ZF ancestor, and a Zap1/ZafA ancestor in the early fungi; a Zfp106 ancestor in early bilaterians; a Twincl ancestor in the founder of *Aspergillus *and its close siblings; and a Clr1 ancestor in the founder of Dikarya, althogh most Dikarya may have lost the gene. Once the tCWCH2 structures were established, they may have been more strongly conserved in these classes.

### Function and role of tCWCH2

There are several cases that directly indicate the functional importance of the tCWCH2 motif in living organisms. In the case of PacC, mutation of the tryptophan of tCWCH2 induces a loss of its DNA binding activity [31,32]. In case of human ZIC3, the tryptophan mutation is proposed to be associated with the occurrence of a familial congenital heart defect, and biochemical analysis revealed that transcriptional activity and protein stability are decreased in the mutant protein [13,14]. In yeast Zap1, interaction between the two ZF where the tCWCH2 motif is located is required for the high-affinity binding of the zinc ion [45]. Also, it has been shown that the inter-ZF interaction is influenced by the concentration of zinc ion [46], suggesting that the tCWCH2 motif is involved in zinc-sensing in yeast.

Apart from the instances referred to above, the functional significance of the tCWCH2 domain remains unknown. We propose that tCWCH2 may play roles other than the direct DNA binding that is often exerted by classical C2H2, because the linker lengths of tCWCH2 are more highly divergent than those of the canonical C2H2 ZFs, in which linker length is a critical parameter for DNA-C2H2 ZF interactions [60-63]. Furthermore, the structure of GLI, ZIC3, and Zap1 tCWCH2 domains, in which the two adjacent ZFs form a single globular structure [7,13,47], is clearly different from the structure of the DNA-binding C2H2 ZF domain where each globular ZF unit wraps around the major groove of the DNA [7-11,64] (PDBID: GLI, 2GLI; YY1, 1UBD; WT1, 2RPT; Zif268, 1MEY, 1P47; TFIIIA; 1TF3). In the case of GLI tCWCH2, which is the only tCWCH2 with a known DNA-binding 3D structure, the N-terminal ZF of GLI1 tCWCH2 does not contact DNA [7].

Assuming that the tCWCH2 domain has a function other than DNA binding, what roles could tCWCH2 play? Firstly, our domain structure analysis implies that tCWCH2 likely regulates its neighboring domains, such as canonical C2H2 ZF, WD40, and LIL. Zic/Gli/Glis, PacC, Mizf, Aebp2, Zap1/ZafA, Fungl, Clr1, and Fungl-4ZF possess canonical ZF(s) in the C-terminal flanking region of the tCWCH2. In the case of PacC, analyses of CWCH2 motif-containing tryptophan mutants suggests that DNA binding mediated by ZF2 and ZF3 may be influenced by tCWCH2 motifs in ZF1 and ZF2 [32]. It is possible that tCWCH2 motifs in the other members of this group act similarly in the regulation of neighboring canonical C2H2 domains. In transcription factors, DNA binding domains such as the canonical C2H2 ZF, can define a target sequence and fix the protein position (angle, distance, direction) with respect to the DNA. The tCWCH2 motif could provide a 'hinge' or modulatory junction that controls the relative positioning of other functional domains. Secondly, tCWCH2 motifs may provide a capping structure that isolates the structural influence of N-terminal flanking region. In the case of ZIC3, mutations in its tCWCH2 motif increase the random coil contents more than the zinc-free state [13]. A unique terminal structure in a domain composed of repeating structural motifs is also seen in other conserved domains [65-67]. In the case of small leucine-rich repeats and ankyrin repeats, N-capping structures are responsible for protein stability [66,67]. Thirdly, tCWCH2 binding to other molecules is possible, and is observed for many conserved domains. Transcriptional regulator and chromatin remodeling proteins form complexes with many different proteins in order to be functionally active [68,69]. Therefore, the tCWCH2 ZF may not be associated with DNA binding but may serve in protein recognition similar to many C2H2 ZFs [5].

The distribution and conservation of the tCWCH2 sequence motif in various gene classes suggests that this motif has important biological roles. The genes we identified containing one or more tCWCH2 motifs are known to be involved in various biological processes, including chromatin remodeling (Arid2/Rsc9), zinc homeostasis (Zap1/ZafA), pH sensing (PacC), cell cycle regulation, and transcriptional regulation (Zic/Gli/Glis, PacC, Mizf, Aebp2, Zap1/ZafA). In addition, given that several gene classes remain to be functionally characterized (including Fungl, Zfp106, Twincl, Clr1, Fungl-4ZF, *Monosiga *gene, DdHp), the known functional repertoire of the tCWCH2 motif is likely to increase. We propose that the tCWCH2 sequence motif is a widespread and functional protein structural motif and that further structural and functional analyses are required to fully understand its roles in biological processes.

## Conclusions

(1) The 3D structure of tCWCH2 is highly conserved among human ZIC3, human GLI1, and yeast Zap1.

(2) Current databases contained 587 tCWCH2 sequences that can be classified into 11 major gene classes (Zic/Gli/Glis, Arid2/Rsc9, PacC, Mizf, Aebp2, Zap1/ZafA, Fungl, Zfp106, Twincl, Clr1, and Fungl-4ZF) and other minority classes. The tCWCH2 motifs were found in transcription regulatory factors, chromatin remodeling factors, and pH/zinc homeostasis regulating factors.

(3) The tCWCH2 motif is mostly found in Opisthokonta (metazoa, fungi, and choanoflagellates) and Amoebozoa (amoeba, *Dictyostelium discoideum*).

(4) The tCWCH2 motif contains three additional structural features: (i) bias to hydrophobic residues in the ϕ1 and ϕ2 positions, (ii) longer linkers between the two C2H2 motifs, and (iii) insertion of extra sequences. The structural features suggest that the tCWCH2 motif is a specialized motif involved in inter-zinc finger interactions.

## Methods

### Protein structural analysis

Protein structures were obtained from the NCBI (National Center for Biotechnology Information) database as PDB format files and we used iMol http://www.pirx.com/iMol/ and Swiss-PdbViewer http://spdbv.vital-it.ch/ as the PDB viewer applications. The latter was also used for superimposing multiple protein structures. Magic fit options were selected for the superimposition of structure data because the number of amino acid residues was different between proteins. Zif268 (PDBID: 1P47) and TFIIIA (PDBID: 2J7J) were used for the reference structure of the classical C2H2 ZF. For the distance analysis of the neighboring amino acid residues, we used "Neighbors of Selected Residue Side chain" option of the Swiss-PdbViewer. The software showed the list of the neighboring residues with distances. We dealt with the residues if the same residues were detected as the neighbors in more than 10 models of ZIC3 and Zap1 PDB files that include 20 models respectively.

### Database search

We conducted web-based BLAST searches using Pattern Hit Initiated (PHI)-BLAST of NCBI databases http://blast.ncbi.nlm.nih.gov/Blast.cgi. For the sequence pattern of the PHI-BLAST searches, we used "xCxWx(2,35)Cx(5,15)Hx(2,5)Hx(5,25)CxWx(1,3)Cx(5,17)Hx(2,5)Hx". The initial sequence pattern was deduced from the compilation of the Zic family sequences [15,70] and subsequently the intervening sequence lengths were modified to the above pattern until no additional sequences appeared in the search. The seed sequences obtained by PHI-BLAST search were first classified into gene families based on their annotation and on phylogenetic tree analyses (see below). Then a TBLASTN search was performed against (1) all of the following databases: GenBank+RefSeq Nucleotides+EMBL+DDBJ+PDB excluding whole-genome shotgun sequences and expressed sequence tags (*nr *option in the NCBI, TBLASTN search), and (2) whole genome shotgun sequences of the organisms shown in Table 2. The whole genome sequence TBLASTN search was performed using representative sequences for each gene family (Table 3) initially on a eukaryote-wide basis, and then on a metazoa-and-fungus-wide basis. For the metazoa-and-fungus-wide search, sequences closely related to the selected genes were searched in the following gene-species combinations: Arid2/Rsc9, PacC, Zap1/ZafA, Fungl, Twincl, Clr1, and Fungl-4ZF were searched in fungi; and Zic/Gli/Glis, Arid2/Rsc9, Mizf, Aebp2, and Zfp106 were searched in metazoa. The target organisms are listed in Table 2. These organisms were selected on the basis of the following criteria: (1) high genome sequence coverage (>5×), (2) member of a major Eukaryote taxon or considered to be in a phylogenetically important position. The collected sequences were manually checked for the presence of the sequence of the tCWCH2 motif. In addition, we performed protein BLAST searches using representative sequences for 16 gene families (Aebp2, Arid2, Clr1, Csr1, Fungl, Fungl-4ZF, Gli, Glis, Mizf, PacC, Rsc9, Twincl, ZafA, Zap1, Zfp106, Zic) irrespective of the presence of the tCWCH2 motif. These searches revealed the extent of tCWCH2 motif conservation in each gene family. Redundant sequences derived from a single gene in a single species were removed from the collected sequences except for one representative sequence. We obtained 587 non-redundant sequences containing the tCWCH2 sequence motif.

**Table 2:** Eukaryotic organisms used for the TBLASTN search.

Organism		Supergroup		Lower taxon
*Entamoeba*	W	Amoebozoa		Entamoebida
*Dictyostelium*	W	Amoebozoa		Mycetozoa
*Paramecium*	W	Chromalveolata		Ciliata
*Tetrahymena*	W	Chromalveolata		Ciliata
*Guillardia*	W	Chromalveolata		Cryptophyta
*Cryptosporidium*	W	Chromalveolata		Dinoflagella
*Giardia*	W	Excavata		Diplomonadia
*Euglena gracilis*	W	Excavata		Euglenozoa
*Trichomonas vaginalis*	W	Excavata		Parabasalidea
*Monosiga*	W	Opisthokonta		Choanoflagellata
*Batrachochytrium dendrobatidis*	MF	Opisthokonta	Fungi	Chytridiomycota
*Aspergillus*	W	Opisthokonta	Fungi	Dikarya Ascomycota
*Aspergillus fumigatus*	MF	Opisthokonta	Fungi	Dikarya Ascomycota
*Candida*	W	Opisthokonta	Fungi	Dikarya Ascomycota
*Candida albicans*	MF	Opisthokonta	Fungi	Dikarya Ascomycota
*Cryptococcus neoformans*	W	Opisthokonta	Fungi	Dikarya Ascomycota
*Neurospora*	W	Opisthokonta	Fungi	Dikarya Ascomycota
*Neurospora crassa*	MF	Opisthokonta	Fungi	Dikarya Ascomycota
*Saccharomyces*	W	Opisthokonta	Fungi	Dikarya Ascomycota
*Saccharomyces cerevisiae*	MF	Opisthokonta	Fungi	Dikarya Ascomycota
*Schizosaccharomyces*	W	Opisthokonta	Fungi	Dikarya Ascomycota
*Schizosaccharomyces pombe*	MF	Opisthokonta	Fungi	Dikarya Ascomycota
*Yarrowia lipolytica*	MF	Opisthokonta	Fungi	Dikarya Ascomycota
*Cryptococcus neoformans*	MF	Opisthokonta	Fungi	Dikarya Basidiomycota
*Ustilago maydis*	MF	Opisthokonta	Fungi	Dikarya Basidiomycota
*Antonospora locustae*	MF	Opisthokonta	Fungi	Microsporidia
*Encephalitozoon*	W	Opisthokonta	Fungi	Microsporidia
*Encephalitozoon cuniculi*	MF	Opisthokonta	Fungi	Microsporidia
*Rhizopus oryzae*	MF	Opisthokonta	Fungi	Mucoromycotina
*Monosiga brevicollis*	MF	Opisthokonta	Metazoa	Choanoflagellida
*Homo*	W	Opisthokonta	Metazoa	Chordata
*Nematostella*	W	Opisthokonta	Metazoa	Cnidaria
*Nematostella vectensis*	MF	Opisthokonta	Metazoa	Cnidaria; Anthozoa
*Platynereis dumerilii*	MF	Opisthokonta	Metazoa	Cnidaria; Anthozoa
*Hydra magnipapillata*	MF	Opisthokonta	Metazoa	Cnidaria; Hydrozoa
*Hydra vulgaris*	MF	Opisthokonta	Metazoa	Cnidaria; Hydrozoa
*Lottia gigantea*	MF	Opisthokonta	Metazoa	Mollusca; Gastropoda
*Trichoplax*	W	Opisthokonta	Metazoa	Placozoa
*Trichoplax adhaerens*	MF	Opisthokonta	Metazoa	Placozoa; Trichoplax
*Schmidtea mediterranea*	MF	Opisthokonta	Metazoa	Platyhelminthes
*Chlamydomonas*	W	Plantae		Chlorophyta
*Arabidopsis*	W	Plantae		Tracheophyta
*Oryza sativa*	W	Plantae		Tracheophyta
*Bigelowiella*	W	Rhizaria		Cercozoa

Species name and taxa are indicated. W = Eukaryote-wide search, MF = metazoa-and-fungus-wide search.

**Table 3:** Query sequences for the TBLASTN search.

Gene	ID	Organism	Supergroup
AEBP2	EAW96398	*Homo sapiens*	Opisthokonta, Metazoa
ARID2	BAB13383	*Homo sapiens*	Opisthokonta, Metazoa
Clr1	NP_596236	*Schizosaccharomyces pombe*	Opisthokonta, Fungi
DdHp	XP_646336	*Dictyostelium discoideum*	Amoebozoa
Fungl	XP_752394	*Aspergillus fumigatus*	Opisthokonta, Fungi
Fungl	XM_572828	*Cryptococcus neoformans*	Opisthokonta, Fungi
GLI1	1405326A	*Homo sapiens*	Opisthokonta, Metazoa
GLIS2	NP_115964	*Homo sapiens*	Opisthokonta, Metazoa
MIZF	NP_945322	*Homo sapiens*	Opisthokonta, Metazoa
PacC	Q00203	*Aspergillus niger*	Opisthokonta, Fungi
REF6	XP_001771543	*Physcomitrella patens*	Plantae
Rsc9	NP_596197	*Schizosaccharomyces pombe*	Opisthokonta, Fungi
Twincl	XP_751009	*Aspergillus fumigatus*	Opisthokonta, Fungi
Twincl	XP_370514	*Magnaporthe grisea*	Opisthokonta, Fungi
Unclassified	XP_001876539	*Laccaria bicolor*	Opisthokonta, Fungi
ZafA	ABJ98717	*Aspergillus fumigatus*	Opisthokonta, Fungi
Zap1/ZafA	AAD26467	*Candida albicans*	Opisthokonta, Fungi
Zap1/ZafA	XP_001797205	*Phaeosphaeria nodorum*	Opisthokonta, Fungi
Zap1/ZafA	XP_001930819	*Pyrenophora tritici-repentis*	Opisthokonta, Fungi
Zap1	NP_012479	*Saccharomyces cerevisiae*	Opisthokonta, Fungi
ZFP106	NP_071918	*Homo sapiens*	Opisthokonta, Metazoa
ZIC3	NP_003404	*Homo sapiens*	Opisthokonta, Metazoa

For the re-evaluation of the initial PHI-BLAST pattern, we generated a tCWCH2 consensus sequence of "LVCKWDGCSEKLFDSPEELVDHVCEDHVGTQLEYTCLWKGCDRFPFKSRYKLIRHIRSHTGEKP". This sequence was constructed using the following steps: (1) alignment of tCWCH2 by ClustalX software [71], (2) elimination of positions where the frequency of the inserted space "-" was >50%, (3) selection of the amino acid residues most frequently appearing in each position. We performed PHI-BLAST again using four patterns (Additional file 3).

Conserved protein domains in the collected sequences were identified by searching the Conserved Domain Database http://www.ncbi.nlm.nih.gov/Structure/cdd/cdd.shtml that included domains imported from Pfam and SMART databases.

### Sequence analysis

Amino acid sequences of the tCWCH2 motif and one C2H2 ZF in the carboxy flanking were aligned using ClustalX software [71] with default parameter settings. The final adjustment of the alignment was performed manually. For the phylogenic tree analyses, MEGA4 {Neighbor-Joining tree (NJ, JTT model, alpha value was determined by Tree-Puzzle [72]), http://www.megasoftware.net/, [73,74]}, RAxML {Maximal Likelihood (ML, WAG model) tree, http://phylobench.vital-it.ch/raxml-bb/, [75]}, and MrBayes3.1.2 {Bayesian Inference (BI, WAG model) tree, [76]} were used. To generate the consensus sequence figure, the aligned sequence was subjected to a web-based analysis, WebLogo http://weblogo.berkeley.edu/[77]. The PDOC00028 alignment in the Prosite database (12328 sequences, http://au.expasy.org/prosite/) was used for the reference sequence representing the general C2H2 domain. This alignment was entered directly into WebLogo and subjected to linker length analysis. We aligned all of the sequences and then extracted the alignment of each taxon to generate the hierarchically ordered alignment of the consensus sequences. The linker sequence length between the two C2H2 ZF motifs was defined by the number of amino acid residues between the C-terminal histidine residue of the first ZF and the N-terminal cysteine residue of the second ZF.

The PDOC00028 sequence alignment contains the gene name and species together with the start and end position number (e.g., GLI1_HUMAN/301-330). We calculated the linker length by subtracting the end-position number from the start-position number for two ZFs. We excluded linker lengths that were too long (>41), because the average length of general C2H2 sequences (PDOC00028) was 27.95 (n = 12328, standard deviation = 1.39, max. = 42, min. = 10) and the maximum linker length of tCWCH2 was 37.

## List of abbreviations

3D: three-dimensional; tCWCH2: tandem CWCH2; ZF: zinc finger; ZFD: zinc finger domain.

## Authors' contributions

MH carried out the sequence alignment, 3D structure analysis, and statistical analysis. JA conceived and designed the study, and analyzed the data. MH and JA wrote the paper and approved the final manuscript.

## Supplementary Material

Additional file 1 **Neighboring residues of the tryptophans in tCWCH2 3D structure. **(A) Distances of the tCWCH2-forming residues from the tCWCH2 ZF1 tryptophan (left) and the tCWCH2 ZF2 tryptophan (right). Black and red letters indicate residues in the ZF1 and the ZF2 respectively. Gothic letters, zinc chelating residue; *, ϕ1 and ϕ2 in Figure 5 and 6. Note that both tryptophan residues were close to the residues in the other C2H2 ZF unit. (B) Stereo view of the ZIC3, GLI1, and Zap1 tryptophan side chain adjacent residues within 5 Å. Tryptophan residues, zinc chelating histidine, ϕ1, and ϕ2 residues are labeled. For ZIC3 and Zap1, 20 NMR models in wire model are superimposed. For GLI1, cylinder model are shown. White, carbon; red, oxygen; blue, nitrogen; yellow; sulfur. Click here for file

Additional file 2 **List of tandem CWCH2-containing genes.** Gene ID of NCBI or Ensembl http://www.ensembl.org/ or JGI *Lottia gigantea *genome http://genome.jgi-psf.org/Lotgi1/Lotgi1.home.html database and organism names that we collected are listed and categorized according to gene group. The EDV29271 *Trichoplax adhaerens *Zic amino acid sequence was manually added from the genome database, and indicates as "EDV29271+edit". The inset in right bottom indicates the numbers of non-redundant sequences in each class. Click here for file

Additional file 3 **PHI-BLAST pattern validity.** Percentage occurrence of the PHI-BLAST pattern in the tCWCH2 library (A). Most frequent amino acid sequence for tCWCH2 in the PHI-BLAST result. A loose search pattern increases the number of BLAST hits, and increases the percentages of recovery of library (B). The tCWCH2 sequence motif collection contains 551 sequences from the NCBI protein database and 36 sequences from the NCBI nucleotide or other databases. Click here for file

Additional file 4 **The sequence of the conserved domains of Arid2 and Rsc9. **The ARID domain (A) and the ZF domain (B) are conserved in Arid2 and Rsc9. Core amino acid residues of the ARID domain are indicated by asterisks (*) in (A). The structure of the tCWCH2 motifs and flanking sequence are shown in (B). Conserved sequence [IxL (S/T) AxL (I/V) L (K/R) N (I/L) x(K/R)] are indicated by asterisks (*). Hs, *Homo sapiens*; Dr, *Danio rerio*; Bm, *Brugia malayi*; Dm, *Drosophila melanogaster*; Nc *Neurospora crassa*; Spo, *Schizosaccharomyces pombe*. Click here for file

Additional file 5 **Zinc finger domains of Mizf. We found novel ZFs in Mizf. **A pseudo ZF structure was found between ZF4 and ZF5 (indicated as ZF?). Hs, *Homo sapiens*; Dr, *Danio rerio*; Ta, *Trichoplax adhaerens*; Ci, *Ciona intestinalis*; Dm, *Drosophila melanogaster*; Ce, *Caenorhabditis elegans*. Click here for file

Additional file 6 **Sequence alignment of Zap1 and ZafA.** Zinc homeostasis genes Zap1 and ZafA have structural similarity. Conservation of ZF8 was weak. Um, *Ustilago maydis*; Cn, *Cryptococcus neoformans*; Afu, *Aspergillus fumigatus*; Mg, *Magnaporthe grisea*; Yl, *Yarrowia lipolytica*; Ca, *Candida albicans*; Sc, *Saccharomyces cerevisiae*. Click here for file

Additional file 7 **Sequence alignment of zinc finger domain of Fungl, human GLI3, and Fungl-4ZF genes. **We propose the common gene name, Fungl: **Fun**gus **G**li **l**ike. Afu, *Aspergillus fumigatus*; Nc, *Neurospora crassa*; Yl, *Yarrowia lipolytica*; Mgl, *Malasszia globosa*; Um, *Ustilago maydis*; Ro, *Rhizopus oryzae*; Ecu, *Encephalitozoon cuniculi*; Ca, *Candida albicans*; Lbi, *Laccaria bicolor*; Eb, *Enterocytozoon bieneusi*; Alo, *Antonospora locustae*; Cn, *Cryptococcus neoformans*; Hs, *Homo sapiens*; Bde, *Batrachochytrium dendrobatidis*. Click here for file

Additional file 8 **Sequence alignment of Twincl zinc finger domain**. Based on its structural features, we named this gene family Twincl (**T**andem C**W**CH2 prote**in C**-termina**l)**. Afu, *Aspergillus fumigatus*; Nf, *Neosartorya fischeri*; Cim, *Coccidioides immitis*; Pn, *Phaeosphaeria nodorum*; Gz, *Gibberella zeae*; Mgr, *Magnaporthe grisea*; Nc, *Neurospora crassa*. Click here for file

Additional file 9 **Sequence alignment of Clr1 zinc finger domain.** Spo, *Schizosaccharomyces pombe*; Sj, *Schizosaccharomyces japonicus*; Cn, *Cryptococcus neoformans*. Click here for file

Additional file 10 **Sequence alignment of *Monosiga*, *Dictyostelium*, and plant gene. **(A) Zinc finger domain alignment of *Dictyostelium discoideum *hypothetical protein (DdHp) and *Monosiga brevicollis *tCWCH2. DdHp2 contains two tCWCH2 domains {ZF12 (ZF1-ZF2) and ZF34 (ZF3-ZF4)}. (B) Plant REF6 *Physcomitrella patens *(XP_001771543) gene has tCWCH2 sequence. It has strong homology to *Arabidopsis *REF6 (NP_680116), *Oryza sativa *gene (NP_001045137) and *Vitis vinifera *(CAO64178) gene, but the tryptophan residue is not conserved in *Arabidopsis *and other plants. An alignment of zinc finger domain of these genes is shown. Amino acid identity between sequences is shown by a dot (.). Click here for file

Additional file 11 **Sequence alignment for phylogenic tree analysis. **Conserved cysteine and histidine residues are indicated with an asterisk (*). Afr, *Artemia franciscana*; Afu, *Aspergillus fumigatus*; Aga, *Anopheles gambiae*; Alo, *Antonospora locustae*; Ape, *Asterina pectinifera*; Bde, *Batrachochytrium dendrobatidis*; Bfl, *Branchiostoma floridae*; Cin, *Ciona intestinalis*; Cn, *Cryptococcus neoformans*; Co, *Corbicula *sp.; Da, *Dicyema acuticephalum*; Dd, *Dictyostelium discoideum*; Dj, *Dugesia japonica*; Dm, *Drosophila melanogaster*; Hr, *Halocynthia roretzi*; Hs, *Homo sapiens*; Hv, *Hydra vulgaris*; Lbl, *Loligo bleekeri*; Mms, *Mus musculus*; Nf, *Neosartorya fischeri*; Nve, *Nematostella vectensis*; Oo, *Octopus ocellatus*; Pi, *Pandinus imperator*; Ro, *Rhizopus oryzae*; Sc, *Saccharomyces cerevisiae*; Sm, *Schistosoma mansoni*; Sp, *Strongylocentrotus purpuratus*; Sso, *Spisula solidissima*; Ssu, *Scolionema suvaense*; Ta, *Trichoplax adhaerens*; Tt, *Tubifex tubifex*; Um, *Ustilago maydis*; Xl, *Xenopus laevis*; Yl, *Yarrowia lipolytica*. Click here for file

Additional file 12 **BI tree of tCWCH2+1ZF.** BI tree analysis was performed on 8 × 10^6 ^generations using WAG model. Average standard deviation of split frequencies was lower than 0.001 at the end of the analysis. Abbreviations are the same as those in Additional file 11. Click here for file

Additional file 13 **ML tree of tCWCH2+1ZF. **ML tree analysis was performed using WAG model with "empirical base frequencies", "maximum likelihood search", and "estimate proportion of invariable sites" options of RAxML [75]. 100 replicates were set for the bootstrap analysis. Abbreviations are the same as those in Additional file 11. Click here for file

Additional file 14 **NJ tree of tCWCH2+1ZF. **NJ tree analysis was performed using JTT model and "pair wise deletion", "Rate among site = different (gamma distribution = 0.62)" options of MEGA4 (http://www.megasoftware.net/, [73,74]). The gamma distributions alpha parameter was calculated by "Tree-Puzzle" (http://www.tree-puzzle.de/, [72]). 1000 replicates were set for the bootstrap analysis. Abbreviations are the same as those in Additional file 11. Click here for file
