# Supplementary material for: Characterization of the tandem CWCH2 sequence motif: a hallmark of inter-zinc finger interactions
Source: BMC Evol Biol. 2010 Feb 19;10:53. doi: 10.1186/1471-2148-10-53 (PMC2837044; doi:10.1186/1471-2148-10-53)

A

	ZF1		
Distance	ZIC3 (W255)	GLI1 (W239)	Zap1 (W583)
3Å	H286	H260	H604
4Å	C268<br>V282*<br>H286<br>V287<br>C297	C237<br>H271<br>W272<br>M292*	C581<br>C586<br>L600*<br>N619<br>W620
5Å	C253<br>D257<br>Y298<br>W299<br>I319*	C242<br>I256*<br>I261<br>C270<br>L288	

	ZF2		
Distance	ZIC3 (W299)	GLI1 (W272)	Zap1 (W620)
3Å	H322	R238	H641
4Å	V282*<br>C297<br>C302<br>H322	C237<br>W239<br>C270<br>M292*<br>H295	K582<br>W583<br>L600*<br>C618<br>C623<br>I637*
5Å	C253<br>K254<br>W255<br>E300<br>I319	L252<br>I256*<br>C275<br>T296	E621

B

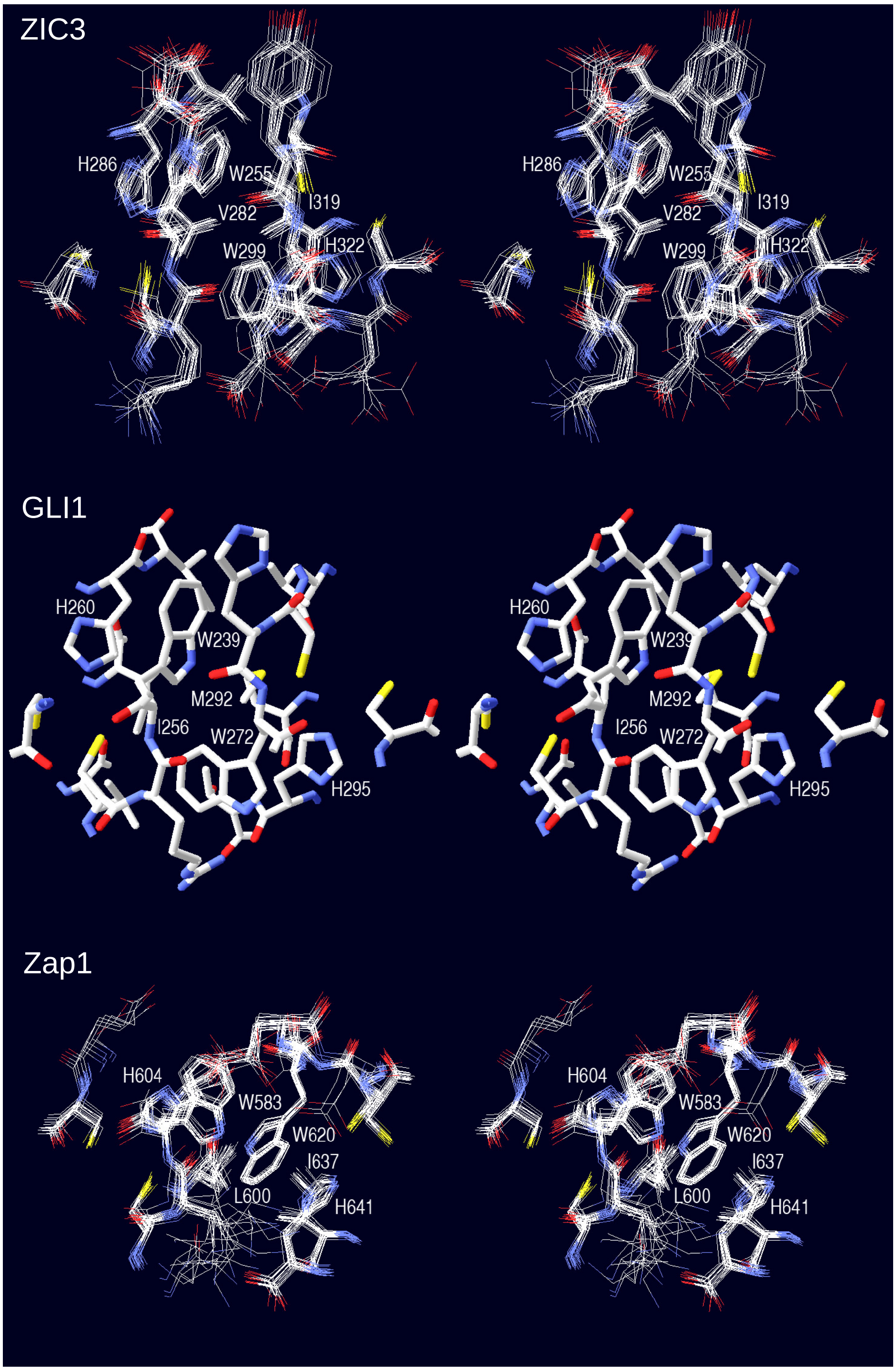

Supplement: Additional file 1 — Neighboring residues of the tryptophans in tCWCH2 3D structure. (A) Distances of the tCWCH2-forming residues from the tCWCH2 ZF1 tryptophan (left) and the tCWCH2 ZF2 tryptophan (right). Black and red letters indicate residues in the ZF1 and the ZF2 respectively. Gothic letters, zinc chelating residue; *, ϕ1 and ϕ2 in Figure 5 and 6. Note that both tryptophan residues were close to the residues in the other C2H2 ZF unit. (B) Stereo view of the ZIC3, GLI1, and Zap1 tryptophan side chain adjacent residues within 5 Å. Tryptophan residues, zinc chelating histidine, ϕ1, and ϕ2 residues are labeled. For ZIC3 and Zap1, 20 NMR models in wire model are superimposed. For GLI1, cylinder model are shown. White, carbon; red, oxygen; blue, nitrogen; yellow; sulfur. [file 1471-2148-10-53-S1.PDF]
