# Supplementary material for: Characterization of the tandem CWCH2 sequence motif: a hallmark of inter-zinc finger interactions
Source: BMC Evol Biol. 2010 Feb 19;10:53. doi: 10.1186/1471-2148-10-53 (PMC2837044; doi:10.1186/1471-2148-10-53)
Supplement: Additional file 2 — List of tandem CWCH2-containing genes. Gene ID of NCBI or Ensembl http://www.ensembl.org/ or JGI Lottia gigantea genome http://genome.jgi-psf.org/Lotgi1/Lotgi1.home.html database and organism names that we collected are listed and categorized according to gene group. The EDV29271 Trichoplax adhaerens Zic amino acid sequence was manually added from the genome database, and indicates as "EDV29271+edit". The inset in right bottom indicates the numbers of non-redundant sequences in each class. [file 1471-2148-10-53-S2.PDF]

Zic/Gli/Glis	
ABRM01011905	*Hydra magnipapillata*
DN245523	*Hydra magnipapillata*
AAR10817	*Hydra vulgaris*
ABM67338	*Hylobates klossii*
ABM22385	*Ictalurus punctatus*
AAD08922	*Junonia coenia*
BAE94135	*Loligo bleekeri*
ABU48965	*Lophius americanus*
fgenesh2_pg.C_sca_51000043	*Lottia gigantea*
fgenesh2_pg.C_sca_30000009	*Lottia gigantea*
fgenesh2_pg.C_sca_42000024	*Lottia gigantea*
fgenesh2_pg.C_sca_62000020	*Lottia gigantea*
ABM22388	*Lutjanus mahogoni*
ABM22387	*Lycodes terraenovae*
XP_001093759	*Macaca mulatta*
XP_001110770	*Macaca mulatta*
XP_001116072	*Macaca mulatta*
XP_001098108	*Macaca mulatta*
XP_001106844	*Macaca mulatta*
XP_001083553	*Macaca mulatta*
ABU48973	*Masturus lanceolatus*
BAE54350	*Molgula tectiformis*
BAE54349	*Molgula tectiformis*
XP_001376758	*Monodelphis domestica*
XP_001363758	*Monodelphis domestica*
XP_001371524	*Monodelphis domestica*
XP_001376775	*Monodelphis domestica*
XP_001365298	*Monodelphis domestica*
XP_001371069	*Monodelphis domestica*
XP_001369348	*Monodelphis domestica*
ABU48935	*Morone chrysops*
P46684	*Mus musculus*
BAC34504	*Mus musculus*
Q62521	*Mus musculus*
Q61467	*Mus musculus*
EDL00627	*Mus musculus*
P47806	*Mus musculus*
AAH82604	*Mus musculus*
NP_032156	*Mus musculus*
AAH66157	*Mus musculus*
NP_112461	*Mus musculus*
Q6XP49	*Mus musculus*
XP_001605506	*Nasonia vitripennis*
XP_001602427	*Nasonia vitripennis*
XP_001602003	*Nasonia vitripennis*
XP_001605160	*Nasonia vitripennis*
XM_001631983	*Nematostella vectensis*
BAE94126	*Nematostella vectensis*
XP_001628007	*Nematostella vectensis*
XP_001628006	*Nematostella vectensis*
XM_001627935	*Nematostella vectensis*
XM_001629586	*Nematostella vectensis*
XP_001636498	*Nematostella vectensis*
XP_001637240	*Nematostella vectensis*
BAE94136	*Octopus ocellatus*
ABU48968	*Ogcocephalus parvus*
ABM22380	*Oncorhynchus mykiss*
ABU48966	*Oneirodes macrosteus*
ABM22384	*Oreochromis niloticus*
XP_001507901	*Ornithorhynchus anatinus*
XP_001506695	*Ornithorhynchus anatinus*
XP_001520378	*Ornithorhynchus anatinus*
XP_001514813	*Ornithorhynchus anatinus*
BAC78801	*Oryzias latipes*
BAC78800	*Oryzias latipes*
ABM54360	*Pan paniscus*
XP_516806	*Pan troglodytes*
XP_526339	*Pan troglodytes*
XP_001158280	*Pan troglodytes*
NP_001029362	*Pan troglodytes*
XP_001137126	*Pan troglodytes*
BAE94137	*Pandinus imperator*
ABM87883	*Papio hamadryas*
ABW76119	*Petromyzon marinus*
ABU48938	*Platax boersii*
ABU48939	*Platax teira*
ABM89385	*Pongo pygmaeus*
AAS91567	*Pristionchus pacificus*
NP_073168	*Rattus norvegicus*
NP_001101862	*Rattus norvegicus*
EDL86160	*Rattus norvegicus*
EDL77543	*Rattus norvegicus*
NP_001101861	*Rattus norvegicus*
XP_345833	*Rattus norvegicus*
NP_001100639	*Rattus norvegicus*
XP_001054276	*Rattus norvegicus*
EDL90430	*Rattus norvegicus*
NP_001100448	*Rattus norvegicus*
EDM13072	*Rattus norvegicus*
ABU48971	*Rhinecanthus aculeatus*
ABM47700	*Saguinus labiatus*
AAV74255	*Saimiri boliviensis*
ABU48958	*Scatophagus argus*
AAX26340	*Schistosoma japonicum*
BAE94122	*Schistosoma mansoni*
CD196065	*Schistosoma mansoni*
AAWT01003419	*Schmidtea mediterranea*
AAWT01028541	*Schmidtea mediterranea*
DN296744	*Schmidtea mediterranea*
BAE94143	*Scolionema suvaense*
ABU48959	*Selenotoca multifasciata*
ABM22390	*Semotilus atromaculatus*
ABU48960	*Siganus doliatus*
ABU48963	*Siganus vulpinus*
ABU48974	*Sphoeroides dorsalis*
BAE94123	*Spisula solidissima*
XP_783842	*Strongylocentrotus purpuratus*
XP_785526	*Strongylocentrotus purpuratus*
XP_798511	*Strongylocentrotus purpuratus*
CAG04272	*Tetraodon nigroviridis*
CAG07295	*Tetraodon nigroviridis*
CAG09820	*Tetraodon nigroviridis*
CAG04274	*Tetraodon nigroviridis*
CAG12320	*Tetraodon nigroviridis*
CAG11206	*Tetraodon nigroviridis*
CAG00860	*Tetraodon nigroviridis*
CAF96507	*Tetraodon nigroviridis*
CAG05504	*Tetraodon nigroviridis*
CAG01444	*Tetraodon nigroviridis*
CAG00166	*Tetraodon nigroviridis*
CAF99221	*Tetraodon nigroviridis*
ABU48969	*Triacanthodes anomalus*
XP_968410	*Tribolium castaneum*
XP_970110	*Tribolium castaneum*
XP_969964	*Tribolium castaneum*
EDV29271+edit	*Trichoplax adhaerens*
ABU48970	*Trixiphichthys weberi*
BAE94130	*Tubifex tubifex*
O73689	*Xenopus laevis*
NP_001079428	*Xenopus laevis*
AAH57699	*Xenopus laevis*
NP_001121252	*Xenopus laevis*
NP_001079126	*Xenopus laevis*
Q91690	*Xenopus laevis*
NP_001081894	*Xenopus laevis*
NP_001081440	*Xenopus laevis*
NP_001081442	*Xenopus laevis*
Q98T94	*Xenopus laevis*
NP_001017025	*Xenopus tropicalis*
NP_001005691	*Xenopus tropicalis*
ABU48948	*Zebrasoma scopas*

Arid2/Rsc9	
XP_001945762	*Acyrthosiphon pisum*
XP_001653162	*Aedes aegypti*
XP_001538064	*Ajellomyces capsulatus*
XP_308780	*Anopheles gambiae*
XP_001120053	*Apis mellifera*
XP_001267933	*Aspergillus clavatus*
XP_750672	*Aspergillus fumigatus*
XP_659533	*Aspergillus nidulans*
XP_001394268	*Aspergillus niger*
XP_001819382	*Aspergillus oryzae*
XP_001215060	*Aspergillus terreus*
XP_596055	*Bos taurus*
XP_001556405	*Botryotinia fuckeliana*
XP_001895110	*Brugia malayi*
XP_001678539	*Caenorhabditis briggsae*
NP_495679	*Caenorhabditis elegans*
XP_001227124	*Chaetomium globosum*
AAZ14906	*Coprinellus disseminatus*
XP_001829164	*Coprinopsis cinerea*
XP_569068	*Cryptococcus neoformans*
XP_001843910	*Culex quinquefasciatus*
NP_001071231	*Danio rerio*
XP_001961319	*Drosophila ananassae*
XP_001970758	*Drosophila erecta*
XP_001994976	*Drosophila grimshawi*
NP_610216	*Drosophila melanogaster*
XP_002006823	*Drosophila mojavensis*
XP_002026330	*Drosophila persimilis*
XP_001361964	*Drosophila pseudoobscura*
XP_002032613	*Drosophila sechellia*
XP_002080300	*Drosophila simulans*
XP_002049205	*Drosophila virilis*
XP_002062748	*Drosophila willistoni*
XP_002089345	*Drosophila yakuba*
XP_001489660	*Equus caballus*
XP_416046	*Gallus gallus*
XP_389671	*Gibberella zeae*
NP_689854	*Homo sapiens*
XP_001874007	*Laccaria bicolor*
fgenesh2_pg.C_sca_101000043	*Lottia gigantea*
XP_001092151	*Macaca mulatta*
XP_365791	*Magnaporthe grisea*
XP_001729202	*Malassezia globosa*
XP_001367064	*Monodelphis domestica*
NP_780460	*Mus musculus*
XP_001605590	*Nasonia vitripennis*
XP_001257899	*Neosartorya fischeri*
XP_957531	*Neurospora crassa*
XP_001506053	*Ornithorhynchus anatinus*
XP_001804530	*Phaeosphaeria nodorum*
XP_001906137	*Podospora anserina*
XP_001931820	*Pyrenophora tritici-repentis*
XP_345868	*Rattus norvegicus*
AAX28273	*Schistosoma japonicum*
NP_596197	*Schizosaccharomyces pombe*
XP_001596218	*Sclerotinia sclerotiorum*
CAF94469	*Tetraodon nigroviridis*
XP_975398	*Tribolium castaneum*
XP_002111154	*Trichoplax adhaerens*
XP_761409	*Ustilago maydis*
AAI60686	*Xenopus laevis*

Aebp2	
XP_001654760	*Aedes aegypti*
XP_392199	*Apis mellifera*
NP_001076954	*Bos taurus*
EEA60137	*Branchiostoma floridae*
XP_543783	*Canis familiaris*
NP_001071973	*Ciona intestinalis*
XP_001861251	*Culex quinquefasciatus*
NP_998659	*Danio rerio*
XP_001961324	*Drosophila ananassae*
XP_001970761	*Drosophila erecta*
XP_001994972	*Drosophila grimshawi*
AAK49526	*Drosophila melanogaster*
XP_002006827	*Drosophila mojavensis*
XP_002026323	*Drosophila persimilis*
XP_002138977	*Drosophila pseudoobscura*
XP_002032608	*Drosophila sechellia*
XP_002080295	*Drosophila simulans*
XP_002049201	*Drosophila virilis*
XP_002062805	*Drosophila willistoni*
XP_002089341	*Drosophila yakuba*
XP_001916199	*Equus caballus*
XP_416415	*Gallus gallus*
EAW96398	*Homo sapiens*
ABRM01023703	*Hydra magnipapillata*
fgenesh2_pg.C_sca_11000282	*Lottia gigantea*
XP_001096639	*Macaca mulatta*
XP_001366237	*Monodelphis domestica*
AAH25467	*Mus musculus*
XP_001604287	*Nasonia vitripennis*
XM_001633166	*Nematostella vectensis*
XP_001160744	*Pan troglodytes*
NP_001100096	*Rattus norvegicus*
AAW26940	*Schistosoma japonicum*
AAWT01013938	*Schmidtea mediterranea*
XP_796561	*Strongylocentrotus purpuratus*
CAF96765	*Tetraodon nigroviridis*
XP_969770	*Tribolium castaneum*
EDV26905	*Trichoplax adhaerens*
NP_001085340	*Xenopus laevis*

PacC	
Q96X49	*Acremonium chrysogenum*
XP_001538988	*Ajellomyces capsulatus*
NP_985737	*Ashbya gossypii*
XP_001271195	*Aspergillus clavatus*
EDP52555	*Aspergillus fumigatus*
Q5XL24	*Aspergillus giganteus*
XP_660459	*Aspergillus nidulans*
Q00203	*Aspergillus niger*
BAB20756	*Aspergillus oryzae*
Q96UW0	*Aspergillus parasiticus*
AATT01000024	*Batrachochytrium dendrobatidis*
AAV54519	*Botryotinia fuckeliana*
AAD51714	*Candida albicans*
Q873Y3	*Candida dubliniensis*
XP_445850	*Candida glabrata*
XP_001221899	*Chaetomium globosum*
XP_001244284	*Coccidioides immitis*
XP_001829114	*Coprinopsis cinerea*
XM_767384	*Cryptococcus neoformans*
XP_458676	*Debaryomyces hansenii*
Q00202	*Emericella nidulans*
ACB12920	*Exophiala dermatitidis*
Q870A3	*Fusarium oxysporum*
Q8J1U9	*Gibberella fujikuroi*
Q873X0	*Gibberella moniliformis*
ABL96218	*Glomerella acutata*
ABR37627	*Glomerella cingulata*
ACD63077	*Humicola grisea*
ABK60115	*Hypocrea lixii*
XP_453982	*Kluyveromyces lactis*
XP_001525711	*Lodderomyces elongisporus*
Q52B93	*Magnaporthe grisea*
XP_001263266	*Neosartorya fischeri*
XP_957214	*Neurospora crassa*
Q8J257	*Paracoccidioides brasiliensis*
Q01864	*Penicillium chrysogenum*
XP_001801978	*Phaeosphaeria nodorum*

PacC	
XP_001483038	*Pichia guilliermondii*
XP_001388010	*Pichia stipitis*
XP_001908286	*Podospora anserina*
XP_001930424	*Pyrenophora tritici-repentis*
AACW02000025	*Rhizopus oryzae*
CAA51462	*Saccharomyces cerevisiae*
AAF93178	*Sclerotinia sclerotiorum*
Q9C1A4	*Trichophyton rubrum*
CAG34353	*Ustilago maydis*
XP_001642822	*Vanderwaltozyma polyspora*
XP_500847	*Yarrowia lipolytica*

Mizf	
XP_001951026	*Acyrthosiphon pisum*
XP_001647925	*Aedes aegypti*
XP_311985	*Anopheles gambiae*
NP_001033620	*Bos taurus*
EEA53083	*Branchiostoma floridae*
XP_001896483	*Brugia malayi*
XP_001669460	*Caenorhabditis briggsae*
NP_493579	*Caenorhabditis elegans*
XP_853239	*Canis familiaris*
XP_002121903	*Ciona intestinalis*
NP_001017764	*Danio rerio*
XP_001964031	*Drosophila ananassae*
XP_001982411	*Drosophila erecta*
XP_001992178	*Drosophila grimshawi*
NP_652054	*Drosophila melanogaster*
XP_002011528	*Drosophila mojavensis*
XP_002022661	*Drosophila persimilis*
XP_001355138	*Drosophila pseudoobscura*
XP_002040250	*Drosophila sechellia*
XP_002105834	*Drosophila simulans*
XP_002059512	*Drosophila virilis*
XP_002070851	*Drosophila willistoni*
XP_002099694	*Drosophila yakuba*
XP_001917467	*Equus caballus*
XP_417894	*Gallus gallus*
NP_945322	*Homo sapiens*
ABRM01017523	*Hydra magnipapillata*
fgenesh2_pg.C_sca_85000130	*Lottia gigantea*
Q8K1K9	*Macaca mulatta*
EDL25575	*Mus musculus*
XP_001606774	*Nasonia vitripennis*
XP_001621526	*Nematostella vectensis*
XP_001514126	*Ornithorhynchus anatinus*
ENSPTRT00000008114	*Pan troglodytes*
EDL95290	*Rattus norvegicus*
CAG02871	*Tetraodon nigroviridis*
XP_971217	*Tribolium castaneum*
EDV26193	*Trichoplax adhaerens*
NP_001086079	*Xenopus laevis*
NP_001086640	*Xenopus tropicalis*

Zap1/ZafA	
XP_001541262	*Ajellomyces capsulatus*
NP_984687	*Ashbya gossypii*
XP_001269254	*Aspergillus clavatus*
ABJ98717	*Aspergillus fumigatus*
XP_658869	*Aspergillus nidulans*
XP_001392305	*Aspergillus niger*
XP_001825350	*Aspergillus oryzae*
XP_001210264	*Aspergillus terreus*
XP_001556845	*Botryotinia fuckeliana*
AAD26467	*Candida albicans*
XP_447926	*Candida glabrata*
XP_001239281	*Coccidioides immitis*
XP_001831390	*Coprinopsis cinerea*
XP_772579	*Cryptococcus neoformans*
CAR65554	*Debaryomyces hansenii*
XP_381198	*Gibberella zeae*
XP_455146	*Kluyveromyces lactis*
XP_001875215	*Laccaria bicolor*
XP_001524482	*Lodderomyces elongisporus*
XP_362011	*Magnaporthe grisea*
XP_001732503	*Malassezia globosa*
XP_001264761	*Neosartorya fischeri*
CAE76512	*Neurospora crassa*
XP_001797205	*Phaeosphaeria nodorum*
XP_001484395	*Pichia guilliermondii*
XP_001387717	*Pichia stipitis*
XP_001930819	*Pyrenophora tritici-repentis*
AACW02000306	*Rhizopus oryzae*
NP_012479	*Saccharomyces cerevisiae*
XP_001592596	*Sclerotinia sclerotiorum*
XP_756411	*Ustilago maydis*
XP_001645067	*Vanderwaltozyma polyspora*
XP_503203	*Yarrowia lipolytica*

Fungl	
XP_001541278	*Ajellomyces capsulatus*
DR043952	*Antonospora locustae*
XP_001269235	*Aspergillus clavatus*
XP_752394	*Aspergillus fumigatus*
XP_658858	*Aspergillus nidulans*
XP_001392336	*Aspergillus niger*
XP_001825327	*Aspergillus oryzae*
XP_001210304	*Aspergillus terreus*
XP_001558403	*Botryotinia fuckeliana*
XP_721460	*Candida albicans*
XP_001239290	*Coccidioides immitis*
XM_572828	*Cryptococcus neoformans*
XP_457736	*Debaryomyces hansenii*
NP_585871	*Encephalitozoon cuniculi*
ABGB01000087	*Enterocytozoon bieneusi*
XP_380653	*Gibberella zeae*
XP_001885936	*Laccaria bicolor*
XP_001526781	*Lodderomyces elongisporus*
XP_363323	*Magnaporthe grisea*
XP_001732339	*Malassezia globosa*
XP_001264743	*Neosartorya fischeri*
XP_965306	*Neurospora crassa*
XP_001797179	*Phaeosphaeria nodorum*
XP_001487806	*Pichia guilliermondii*
XP_001383735	*Pichia stipitis*
XP_001908562	*Podospora anserina*
XP_001930831	*Pyrenophora tritici-repentis*
AACW02000043	*Rhizopus oryzae*
CAB59682	*Schizosaccharomyces pombe*
XP_001597005	*Sclerotinia sclerotiorum*
XP_759845	*Ustilago maydis*
XP_505759	*Yarrowia lipolytica*

Zfp106	
XP_001255518	*Bos taurus*
EEA71173	*Branchiostoma floridae*
XP_859468	*Canis familiaris*
XP_002126391	*Ciona intestinalis*
XP_001503318	*Equus caballus*
XP_421158	*Gallus gallus*
NP_071918	*Homo sapiens*
fgenesh2_pg.C_sca_89000068	*Lottia gigantea*
XP_001103703	*Macaca mulatta*
XP_001362420	*Monodelphis domestica*
O88466	*Mus musculus*
XP_523059	*Pan troglodytes*
EDL79950	*Rattus norvegicus*
XP_001199532	*Strongylocentrotus purpuratus*
CAF91116	*Tetraodon nigroviridis*
CAJ83853	*Xenopus tropicalis*

Twincl	
XP_001536568	*Ajellomyces capsulatus*
XP_001268223	*Aspergillus clavatus*
XP_751009	*Aspergillus fumigatus*
XP_001392864	*Aspergillus nidulans*
XP_658173	*Aspergillus nidulans*
XP_001821029	*Aspergillus oryzae*
XP_001214221	*Aspergillus terreus*
XP_001240170	*Coccidioides immitis*
XP_384555	*Gibberella zeae*
XP_370514	*Magnaporthe grisea*
XP_001258243	*Neosartorya fischeri*
XP_956474	*Neurospora crassa*
XP_001797269	*Phaeosphaeria nodorum*

Clr1	
XP_772105	*Cryptococcus neoformans*
XP_775775	*Cryptococcus neoformans*
XP_002172202	*Schizosaccharomyces japonicus*
NP_596236	*Schizosaccharomyces pombe*

Fungus Gli like 4 ZF	
EE009677	*Rhizopus oryzae*
AATT01000065	*Batrachochytrium dendrobatidis*

Dictyostelium	
XM_634128	*Dictyostelium discoideum*
XM_641244	*Dictyostelium discoideum*

Monosiga	
XM_001747841	*Monosiga brevicollis*

Unclassified sequences		
XP_567439	*Cryptococcus neoformans*	PacC like
XP_001876539	*Laccaria bicolor*	Fungus Gli like?
XP_001879153	*Laccaria bicolor*	?
CAD21133	*Neurospora crassa*	?
AACW0200030[illegible]	*Rhizopus oryzae*	Gli like ZF?
AAX28296	*Schistosoma japonicum*	Mizf like

Isolated tCWCH2 sequences in other gene families		
XP_001656835	*Aedes aegypti*	Sp8
XP_317797	*Anopheles gambiae*	Sp8?
XP_001895421	*Brugia malayi*	pbrm-1
XP_001846878	*Culex quinquefasciatus*	Sp8?
XP_001639783	*Nematostella vectensis*	Kruppel-like factor 5
XP_001911713	*Podospora anserina*	TPR2

Plant		
AK110182	*Oryza sativa*	contamination?
XP_001771543	*Physcomitrella patens*	ELF6

Gene class	n
Zic/Gli/Glis	282
Arid2/Rsc9	61
PacC	48
Mizf	40
Aebp2	39
Zap1/ZafA	33
Fungl	32
Zfp106	16
Twincl	13
Clr1	4
Fungl-4ZF	2
Dictyostelium	2
Monosiga	1
Unclassified	6
Isolated tCWCH2	6
Plant	2
Total	587
