# Supplementary material for: Characterization of the tandem CWCH2 sequence motif: a hallmark of inter-zinc finger interactions
Source: BMC Evol Biol. 2010 Feb 19;10:53. doi: 10.1186/1471-2148-10-53 (PMC2837044; doi:10.1186/1471-2148-10-53)
Supplement: Additional file 3 — PHI-BLAST pattern validity. Percentage occurrence of the PHI-BLAST pattern in the tCWCH2 library (A). Most frequent amino acid sequence for tCWCH2 in the PHI-BLAST result. A loose search pattern increases the number of BLAST hits, and increases the percentages of recovery of library (B). The tCWCH2 sequence motif collection contains 551 sequences from the NCBI protein database and 36 sequences from the NCBI nucleotide or other databases. [file 1471-2148-10-53-S3.PDF]

A

PHI pattern	
xCxWx(2,35)Cx(5,15)Hx(2,5)Hx(5,25)CxWx(1,3)Cx(5,17)Hx(2,5)Hx	
pattern match (tCWCH2 amino acid)	617/637 (96.9%)
pattern match (space)	587/637 (92.2%)
total	572/637 (89.8%)

B

LVCKWDGCSEKLFDSPEELVDHVCEDHVGTQLEYTCLWKGCDRFPFKSRYKLIRHIRSHTGEKP

PHI pattern	hit	recover
xCxWx(2,35)Cx(5,15)Hx(2,5)Hx(5,25)CxWx(1,3)Cx(5,17)Hx(2,5)Hx	911	459/551 (83.3%)
xCxWx(2,35)Cx(5,15)Hx(2,5)Hx(5,25)CxWx**(1,6)**Cx(5,17)Hx(2,5)Hx	916	460/551 (83.4%)
xCxWx(2,35)Cx(5,15)Hx(2,5)Hx(5,25)CxWx**(1,6)**Cx**(5,31)**Hx(2,5)Hx	938	463/551 (84.0%)
xCxWx(1,35)Cx(5,15)Hx(2,5)Hx**(5,42)**CxWx**(1,6)**Cx**(5,31)**Hx(2,5)Hx	968	469/551 (85.1%)
