# Supplementary material for: Characterization of the tandem CWCH2 sequence motif: a hallmark of inter-zinc finger interactions
Source: BMC Evol Biol. 2010 Feb 19;10:53. doi: 10.1186/1471-2148-10-53 (PMC2837044; doi:10.1186/1471-2148-10-53)
Supplement: Additional file 4 — The sequence of the conserved domains of Arid2 and Rsc9. The ARID domain (A) and the ZF domain (B) are conserved in Arid2 and Rsc9. Core amino acid residues of the ARID domain are indicated by asterisks (*) in (A). The structure of the tCWCH2 motifs and flanking sequence are shown in (B). Conserved sequence [IxL (S/T) AxL (I/V) L (K/R) N (I/L) x(K/R)] are indicated by asterisks (*). Hs, Homo sapiens; Dr, Danio rerio; Bm, Brugia malayi; Dm, Drosophila melanogaster; Nc Neurospora crassa; Spo, Schizosaccharomyces pombe. [file 1471-2148-10-53-S4.PDF]

A

```
Hs Arid2  FLDELRQFHHSRGSPFKKIPAVGGKE-LDLHGLYTRVTTLGGFAKVSE-KNQWGEIVEEFNFPRS---CSNAAFALKQYYLRYL
Dr Arid2  FLDELRQFHHSRGSPFKKVPVVGGKE-LDLGALYVRVVSLGGFAKVSD-KNQWVELVEDFQFPRS---CSNAAFVLKQYYLRYL
Bm Arid2  FHMNLRTFYKKRWNCRLKSPHIQGVE-VDLFRLYDTVISMGGWQKVSF-NEKWGDIAHAIGLANGV---AVAEHAIKVLYMRQV
Dm Arid2  FWRDLQQFHERRG-TPLTQPARISGKHVDLYKLYNEVTERGGFNKVTM-RDEWDEVYSAMETLRER--CVNGTASIKHIYRRYL
Nc  Rsc9  FIEDLRVFHEKRGTNFDPEPKM-GNLTVDLLKLFKHIVEHGGYDKVSDEKLMWRKMCEGLGLMRHN-APADAYTLKQIFY-KNL
Spo Rsc9  FLSLIESFSQERGVPIDINPKI-GRKPILLYELYKKVIKRGGYDAVSATEDGWTNIAEEFNQSDPAR--S-AGIL-QNVYFKYL
          *      *           *         *  *       **          *                          *   *
```

B

```
Hs Arid2  FMCLWQS-----------------CK--KWFQTPSQVFYHAATEHGGKDVYP-----------G-QCLWEG----
Dr Arid2  FKCLWQA-----------------CK--RWFDTPSQVFYHAATLHGSKDSYP-----------G-QCQWEG----
Bm Arid2  YLCEWNG-----------------CG--KHFSSASFVLYHCTKEHVG-DDH-------S----I-QCHWPR----
Dm Arid2  YICDWRN-----------------CPRKKFKSLNE-LQYHVCSVHCPDHLD---------SDADIYCQWGSGPNF
Nc  Rsc9  LGCQWTRQPTSDEQQQQQQQQQVVCG--AWHLTTESMWNHILTHHLGEAPGTDNKFSNKEGT--YSCHWDS----
Spo Rsc9  QPCRWCLDSGKE------------CG--ELLLGTPLLHSHLQEMHIFPQIL-----------ETGKCRWSD----
                                ZF1

Hs Arid2  CEPFQRQR---FSFIT-----HLQDKHCSKDALLAGL-KQDEPGQAGSQKSSTKQPTVGGTSSTP----------
Dr Arid2  CEPFPRQR---LSFIT-----HLQGKHCSREALIAAL-KLEEQTQGSNPNTSKSPPAAVS-SPPPP---------
Bm Arid2  CDSTVRAK---WSMVT-----HLQDHHCNEAALKAAA-KRRKEGHGLPLGPVN-----------P----------
Dm Arid2  CDNR--------ARKRYSLMTHLIDRHLTTENLRASVQRRLATGIHNVAPTQAPVTIVRNEGHAQRLAGGATGSP
Nc  Rsc9  CR---RYPAP-TKLKLMQLMMHVKT-HLKAEEARHNALYPTLQPSPTPAAGGATEPFPGGGTGGLTTPGGGPVQV
Spo Rsc9  CKYEIQRLTPASELSHYQLLSHIVT-HLHDDS-------------------------------------------
                   ZF2

Hs Arid2  -RAQKAIVNHP----SAALMALRRGSRNLVF-RDFT------DEKEGPITKHIRLTAALILKNIGKYSECG----
Dr Arid2  PRPPKALVNHP----SAALMALRRGSRNLVF-RDFT------DDKEGPVTKHIRLTAALTLKNIAKYSDCG----
Bm Arid2  -ERPREIVQHPGYSKNAAVEAIRRHAFNYL-PRDIT------DDPEGPVTKSIRLTSCLILRNLARYSNDG----
Dm Arid2  SGPASAVPVVGSAAMQAMNRHTTDYTNAKELMDE----------NEGPVTKSIRLTAALIIRNLVTYSATA----
Nc  Rsc9  ASSP-KRSRTRVIKPAGTIS-LTFEETASV--RDERNPNMPPQAAGIP------LSAVLILRNIARNVVKTDAEE
Spo Rsc9  ---LETLVEGRKLSPSREFR-I---------P----LLLTAVDDQGDATGIA--LTATLVLRNLVRSKQGK----
                                                                 **********
```
