# Supplementary material for: Characterization of the tandem CWCH2 sequence motif: a hallmark of inter-zinc finger interactions
Source: BMC Evol Biol. 2010 Feb 19;10:53. doi: 10.1186/1471-2148-10-53 (PMC2837044; doi:10.1186/1471-2148-10-53)
Supplement: Additional file 5 — Zinc finger domains of Mizf. We found novel ZFs in Mizf. A pseudo ZF structure was found between ZF4 and ZF5 (indicated as ZF?). Hs, Homo sapiens; Dr, Danio rerio; Ta, Trichoplax adhaerens; Ci, Ciona intestinalis; Dm, Drosophila melanogaster; Ce, Caenorhabditis elegans. [file 1471-2148-10-53-S5.PDF]

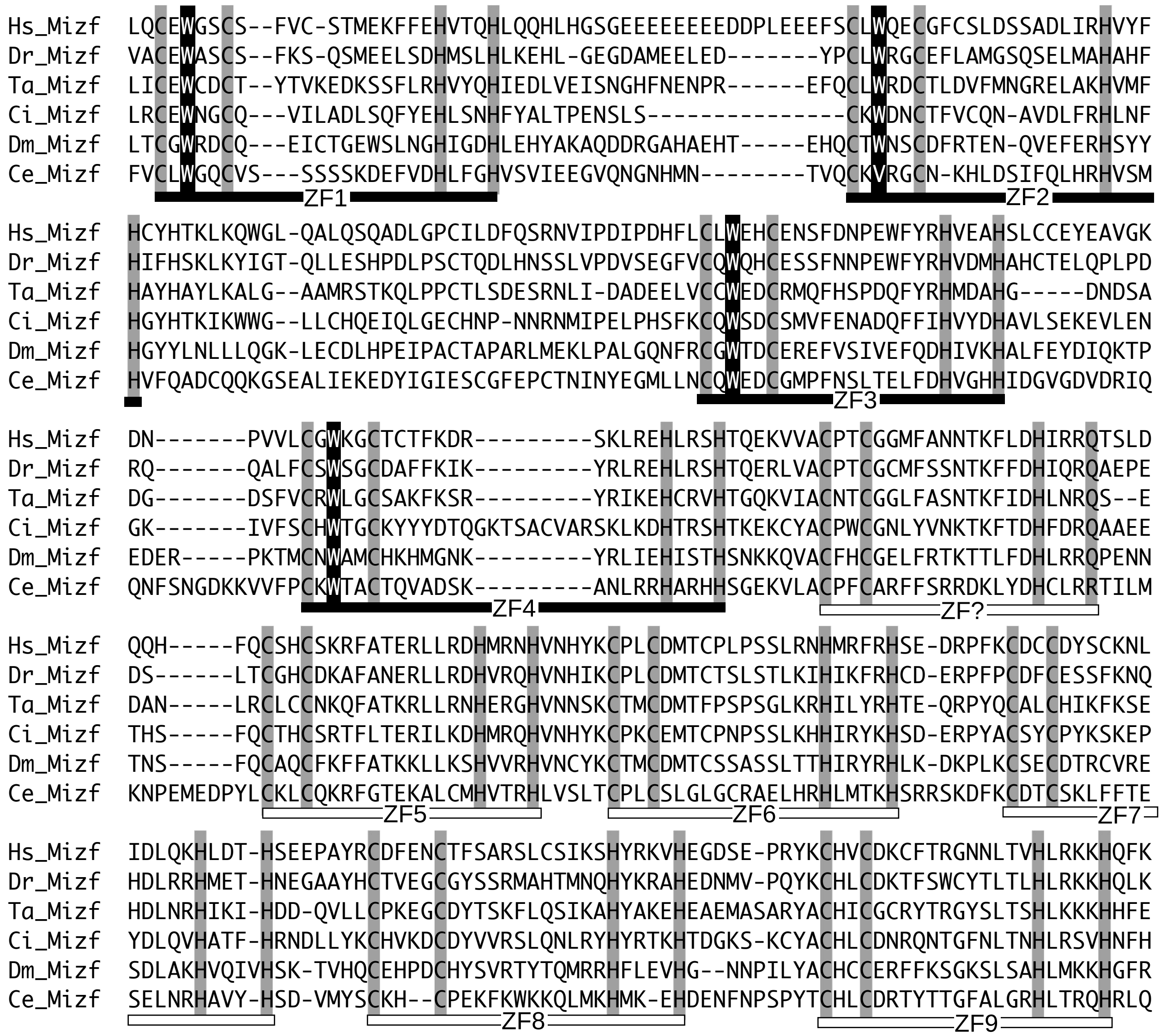
Hs_Mizf LQCEWGSCS--FVC-STMEKFFEHVTQHLQQHLHGSGEEEEEEEEEDDPLEEEFSCLWQECGFCSLDSSADLIRHVYF
Dr_Mizf VACEWASCS--FKS-QSMEELSDHMSLHLKEHL-GEGDAMEELED-------YPCLWRGCEFLAMGSQSELMAHAHF
Ta_Mizf LICEWCDCT--YTVKEDKSSFLRHVYQHIEDLVEISNGHFNENPR------EFQCLWRDCTLDVFMNGRELAKHVMF
Ci_Mizf LRCEWNGCQ---VILADLSQFYEHLSNHFYALTPENSLS---------------CKWDNCTFVCQN-AVDLFRHLNF
Dm_Mizf LTCGWRDCQ---EICTGEWSLNGHIGDHLEHYAKAQDDRGAHAEHT-----EHQCTWNSCDFRTEN-QVEFERHSYY
Ce_Mizf FVCLWGQCVS---SSSSKDEFVDHLFGHVSVIEEGVQNGNHMN--------TVQCKVRGCN-KHLDSIFQLHRHVSM
ZF1
ZF2
Hs_Mizf HCYHTKLKQWGL-QALQSQADLGPCILDFQSRNVIPDIPDHFLCLWEHCENSFDNPEWFYRHVEAHSLCCEYEAVGK
Dr_Mizf HIFHSKLKYIGT-QLLESHPDLPSCTQDLHNSSLVPDVSEGFVCQWQHCESSFNNPEWFYRHVDMHAHCTELQPLPD
Ta_Mizf HAYHAYLKALG--AAMRSTKQLPPCTLSDESRNLI-DADEELVCCWEDCRMQFHSPDQFYRHMDAHG-----DNDSA
Ci_Mizf HGYHTKIKWWG--LLCHQEIQLGECHNP-NNRNMIPELPHSFKCQWSDCSMVFENADQFFIHVYDHAVLSEKEVLEN
Dm_Mizf HGYYLNLLLQGK-LECDLHPEIPACTAPARLMEKLPALGQNFRCGWTDCEREFVSIVEFQDHIVKHALFEYDIQKTP
Ce_Mizf HVFQADCQQKGSEALIEKEDYIGIESCGFEPCTNINYEGMLLNCQWEDCGMPFNSLTELFDHVGHHIDGVGDVDRIQ
ZF3
Hs_Mizf DN-------PVVLCGWKGCTCTFKDR---------SKLREHLRSHTQEKVVACPTCGGMFANNTKFLDHIRRQTSLD
Dr_Mizf RQ-------QALFCSWSGCDAFFKIK---------YRLREHLRSHTQERLVACPTCGCMFSSNTKFFDHIQRQAEPE
Ta_Mizf DG-------DSFVCRWLGCSAKFKSR---------YRIKEHCRVHTGQKVIACNTCGGLFASNTKFIDHLNRQS--E
Ci_Mizf GK-------IVFSCHWTGCKYYYDTQGKTSACVARSKLKDHTRSHTKEKCYACPWCGNLYVNKTKFTDHFDRQAAEE
Dm_Mizf EDER-----PKTMCNWAMCHKHMGNK---------YRLIEHISTHSNKKQVACFHCGELFRTKTTLFDHLRRQPENN
Ce_Mizf QNFSNGDKKVVFPCKWTACTQVADSK---------ANLRRHARHHSGEKVLACPFCARFFSRRDKLYDHCLRRTILM
ZF4
ZF?
Hs_Mizf QQH-----FQCSHCSKRFATERLLRDHMRNHVNHYKCPLCDMTCPLPSSLRNHMRFRHSE-DRPFKCDCCDYSCKNL
Dr_Mizf DS------LTCGHCDKAFANERLLRDHVRQHVNHIKCPLCDMTCTSLSTLKIHIKFRHCD-ERPFPCDFCESSFKNQ
Ta_Mizf DAN-----LRCLCCNKQFATKRLLRNHERGHVNNSKCTMCDMTFPSPSGLKRHILYRHTE-QRPYQCALCHIKFKSE
Ci_Mizf THS-----FQCTHCSRTFLTERILKDHMRQHVNHYKCPKCEMTCPNPSSLKHHIRYKHSD-ERPYACSYCPYKSKEP
Dm_Mizf TNS-----FQCAQCFKFFATKKLLKSHVVRHVNCYKCTMCDMTCSSASSLTTHIRYRHLK-DKPLKCSECDTRCVRE
Ce_Mizf KNPEMEDPYLCKLCQKRFGTEKALCMHVTRHLVSLTCPLCSLGLGCRAELHRHLMTKHSRRSKDFKCDTCSKLFFTE
ZF5
ZF6
ZF7
Hs_Mizf IDLQKHLDT-HSEEPAYRCDFENCTFSARSLCSIKSHYRKVHEGDSE-PRYKCHVCDKCFTRGNNLTVHLRKKHQFK
Dr_Mizf HDLRRHMET-HNEGAAYHCTVEGCGYSSRMAHTMNQHYKRAHEDNMV-PQYKCHLCDKTFSWCYTLTLHLRKKHQLK
Ta_Mizf HDLNRHIKI-HDD-QVLLCPKEGCDYTSKFLQSIKAHYAKEHEAEMASARYACHICGCRYTRGYSLTSHLKKKHHFE
Ci_Mizf YDLQVHATF-HRNDLLYKCHVKDCDYVVRSLQNLRYHYRTKHTDGKS-KCYACHLCDNRQNTGFNLTNHLRSVHNFH
Dm_Mizf SDLAKHVQIVHSK-TVHQCEHPDCHYSVRTYTQMRRHFLEVHG--NNPILYACHCCERFFKSGKSLSAHLMKKHGFR
Ce_Mizf SELNRHAVY-HSD-VMYSCKH--CPEKFKWKKQLMKHMK-EHDENFNPSPYTCHLCDRTYTTGFALGRHLTRQHRLQ
ZF8
ZF9
