# Supplementary material for: Characterization of the tandem CWCH2 sequence motif: a hallmark of inter-zinc finger interactions
Source: BMC Evol Biol. 2010 Feb 19;10:53. doi: 10.1186/1471-2148-10-53 (PMC2837044; doi:10.1186/1471-2148-10-53)
Supplement: Additional file 6 — Sequence alignment of Zap1 and ZafA. Zinc homeostasis genes Zap1 and ZafA have structural similarity. Conservation of ZF8 was weak. Um, Ustilago maydis; Cn, Cryptococcus neoformans; Afu, Aspergillus fumigatus; Mg, Magnaporthe grisea; Yl, Yarrowia lipolytica; Ca, Candida albicans; Sc, Saccharomyces cerevisiae. [file 1471-2148-10-53-S6.PDF]

```
Um  QQCQWGG-------CNKRFWTV-EELVAHVNHSHLARRNATTSAHTAVSDKRVFATTQQEAKSVTHSRAYSSADAQTG
Cn  LSCMWD-------DCFPVPEVPAASSTSHSTFHHYNSDNCQAPHNHQHDHSYAAGEPFNPGTMLRHVLEEHLGIPPDI
Afu GMC-S-GVSA----CPEPHTH--EDQNCHIHLSKLDTRS---------------------------------------
Mg  GPCIVDEPGN-FMFCPFSCVTEHPQL--HNIGITDTTLPQPFFDELFPGLAN------------------------IP
Yl  GNCNYNTDSADDLDCHFVNAHKYD--LNR-YSRHQTACANAMKVYPQEKRPP------------------------VG
Ca  YSCQWEN-------CFKRLNNNTFLN--HVIEDHLEKEEIVK----------------------------------SE
Sc  LKCKWKE-------CPESCSSLFDLQ-RHLLKDHVSQDFKHPMEP---------------------------------
                    ZF1

Um  ATGLECLWKDCHQVAMPVKLEFDTFTPTTEAELWKPNASETVAGHEDEKVSLAILQHLLHDHLGQPS-----------
Cn  IGWPNEAELQAQAQAILEKHHHHHHIDPREALVNHSENCNHVHPHPHSHSHGNSAGTGANDSHPHGHALAHSQFHPNL
Afu TDSFTCFRLPPTPNDSSKASVNIHGNVPLKGPCRSHHRCRIHPHAHVHPYGPYSAYSRQSRSSVSSQLMSSPGETPPP
Mg  DSMKSCHGQG--Y--ASAEQLISHMHACH-------NTILSQYLQGNTTPLLTHSQDPSQIQQYSPTSSSNVAQYMQF
Yl  ISPFQCEWNHCHFRSNDLTEFVSHLMESHQLHTFEHTCSSNNHYPGSFLENVSTMLPLTSTPSSEGSPLSYTPPSTFD
Ca  NSNYQCEWE-CNFTDNDFNSLINHL-KSHQPSNSVETTGFSSLLNNVTNNYALTPLSCTLSNDASPVVALQSPQAPDL
Sc  ---LACNWEDCDFLGDDTCSIVNHINCQHGINFDIQFANPDSFLPGSISKEKHHLLHCPNPQTHEVSKADGAPDMTSA
                 ZF2

Um  ---APPFSLNGAQLLTRSTPSPPLVRPGDTAGHVASSQLTASSSSLWNKKKRKSSNSSSGAYC----SATEKLHCRW-
Cn  HPH--PHSLPHERSYAHSHSLSHSRPLSHEPLPTPPSTVKTEACTSPAASNDSVASTVLTASQ----SSKDL-ICLW-
Afu LEGGASSVLTSPGFSS-------------------------------------------------AEGEVHVCKW-
Mg  QNLPEPIVAHGS-------------SPITTTQNLQANVMTLTPPLTDQSISSPKDGNQIAESNQEQPSNP--NTCLWL
Yl  NDFDSPKYGQMTNPTPSTSSSTSPSVNSAANSAANSAVTSTVASTPPASAAPAPAPAAT------------VHTCQWV
Ca  N-----------------------------------------ITSVKIVPKNKRKSCKPVKKEDQEMDLEHTCNWQ
Sc  NDVSNI--------------------------------------------------------PPIKQPEQVICQWD

Um  ---------MGCSASFDSHS--ALTDHIETEHV-----GSGQAQYECKWIGCARYVSGQKFSQKQK--VLRHIQTHTG
Cn  ---------PGCTIHTPFASTASLMDHLSEMHI-----PKGKDCYTCHWGGCGGEE-GRVFKSRQK--VLRHLQSHIG
Afu -TTTSHGVKRSCGATFADAC--ALQEHLVANHMGTVEGAKG-TGYYCCWEGCHRPD--EPFSQKSK--LQGHFLTHSN
Mg  DDM-----GIPCGHIFEDAS--QLNRHVIEDHL--RYLQKEDNEYLCRWMGCKRQCKTEKRGFPQKSKIERHLQTHTG
Yl  L-EKDP--KHVCKLQFSSAK--DLSDHVIEKHI-----GSRKPEYSCSWDGCERCD--RPFTQRQK--VVRHLQTHTK
Ca  IGTDDNGDPIYCNIKHQSPG--DLHSHLLDVHI-----GSGKHEYHCCWRGCERHNG---KVFTQKQKLIRHIHIHTN
Sc  G----------CNKSFSSAQ--ELNDHLEAVHL-----TRGKSEYQCLWHDCHRTFP-----QRQK--LIRHLKVHSK
              ZF3                                           ZF4

Um  DRPFKCSECGKRFSEQNTLAQHMRTHTLERPYVCDHPGCGKAFSVAGSLTIHRRIHTGSKPFVCTYPGCGKAFAESSN
Cn  HKPFVCGVCNQAFSEAAPLTAHMRRHAQEKPFKCEHPGCGKSFAISSSLTIHMRTHNGEKPFVCPY--CQKGFVEASN
Afu YKNFKCSVCGKLFARQATLERHERSHRGEKPYKCT--ECGKSFTDSSELKTHSRTHTGEKPFKCTFPGCNFQTGDSSN
Mg  DRPFSCPHCPDMFSAKQALEQHILIHKQEKPLKCTFPGCNKSFRQQSARTMHMRVHTKERPLKCNL--CDRTFSESSN
Yl  HRPHQCPVCHYRFAEESVLKQHMRIHSGEKPFQCQ--ICHKTFAASTALSVHMRIHTGEKPLTCKWPGCGKRFSESSN
Ca  FKPCKCDICGASFAVESVLQQHYRVHSGEKPFKCP--ICDKTFATSSSLSIHTRVHTGERPLVCKWPGCNKRFSESSN
Sc  YKPYKCKTCKRCFSSEETLVQHTRTHSGEKPYKCH--ICNKKFAISSSLKIHIRTHTGEKPLQCKI--CGKRFNESSN
                 ZF5                        ZF6                        ZF7

Um  LTKHVRTHTGDKPFKC--DECGKCFSRPDQASRHRK-THERKRGKLGVELPHQDGRAVIS--------------
Cn  LTKHIRTHTGERPFACSHPGCGKKFSRPDQLKRHMT-IHNKPPGEKRRGSGVPAK-------------------
Afu MSSHRLTH-GERKHKCSYPGCSKSFTRPDQLKRHMRTTHKADSTAFPSPSPLSDHFAFPLGTV-----------
Mg  LAKHRRTHAAEGSFHCDFPGCKKTFHRQDQLRRHLK-THAKKVARQASVSQASVSVSPRPSEEPEPESESEMQP
Yl  LTKHMKTHTLEKPFACPHPGCDKRFGRNDQLQRHVK-THEVKEEPEWNAGWQQTVV------------------
Ca  LAKHMRIH--TKKFNC--EACGQSFDKKGVFNRHVL-EHSVEK-------------------------------
Sc  LSKHIKTH--QKKYKCS--DCSKSFDDLGKLNSQKVKCALERKPYL----------------------------
                         ZF8
```
