# Supplementary material for: Characterization of the tandem CWCH2 sequence motif: a hallmark of inter-zinc finger interactions
Source: BMC Evol Biol. 2010 Feb 19;10:53. doi: 10.1186/1471-2148-10-53 (PMC2837044; doi:10.1186/1471-2148-10-53)
Supplement: Additional file 7 — Sequence alignment of zinc finger domain of Fungl, human GLI3, and Fungl-4ZF genes. We propose the common gene name, Fungl: Fungus Gli like. Afu, Aspergillus fumigatus; Nc, Neurospora crassa; Yl, Yarrowia lipolytica; Mgl, Malasszia globosa; Um, Ustilago maydis; Ro, Rhizopus oryzae; Ecu, Encephalitozoon cuniculi; Ca, Candida albicans; Lbi, Laccaria bicolor; Eb, Enterocytozoon bieneusi; Alo, Antonospora locustae; Cn, Cryptococcus neoformans; Hs, Homo sapiens; Bde, Batrachochytrium dendrobatidis. [file 1471-2148-10-53-S7.PDF]

```
Afu_FG   TVCRWEGCNVGDLGNMDDLVQHIHNDH-VG-NRQKR--YSCEWSDCPRKGQTHASGYALRAHMRS-HTREKP
Nc__FG   TVCAWEGCKVGDLGNMDRLVEHIHNSH--IEGRQKK--YTCEWIGCSRKSLPHASGYALKAHMRS-HTREKP
Yl__FG   QICHWVGCET-QVANLDALVDHLNEVH-FG-ARKSK--YQCDWAGCSRQGMVQPSRFALVSHMRS-HTGEKP
Mgl_FG   VRCMWEDCGE-TFNDLQPFIAHLHSFH-IG-IHKSR--YACEWTGCPRKGKSQTSRFALLSHLRS-HTGEKP
Um__FG   VTCQWNDCGE-TFNSLQPFIDHLHNEH-IG-IHKSK--YMCEWTGCIRKGKPQTSRFALLSHLRS-HTGEKP
Ro__FG   -TCQWANCEE-KFDDLQALISHLSEIH-MG-RGKST--YRCEWKDCARIDNPFTKRHKMYAHLRI-HTGERP
Ecu_FG   MRCEWEGCQEEIGDNVR---GHLLS-HI--E--K-DEEARCLWKDCARYGEAQASKHALLAHARR-HTGERP
Ca__FG   FICRWSDCTENTYNNLTSLVNHLNSKHIAQLPPGSNTKYICYWENCARYGLDQPSRFALISHCRT-HTGEKP
Lbi_FG   VTCQWEGCGRVFPHLPP-LIEHIRNDH--VDVQKSS--YTCEWAGCQRGGAQQISRSALISHLRS-HTGEKP
Eb__FG   --CLWANCGKDVSPSD--VQSHINN-H-IEEIKKSAQGLRCRWLNCQY-EKSLSSISNLIVHFKN-HFSPIE
Alo_FG   TVCLWKDCAFECGDSEE-LKKHLET-H------VSAD-LKCCWLGCSRFGEVQPNKYALIAHLRK-HSGDRP
Cn__FG   QKCQWGECQGDFDSKQE-FYGHVKD-H----INAS-KEYACEWRTCSRVGHKQ-GRSLLLTHIRG-HTGERP
Hs_G3    TNCHWEGCAREFDTQEQ-LVHHINNDHIHGE-K---KEFVCRWLDCSREQKPFKAQYMLVVHMRR-HTGEKP
Ro_4ZF   HSCKWESCDAQAPTLDK-LMTHICNSHI-GSGKAT---YHCEWQDCPRNKKPFMKRHKMHNHMRT-HTGERP
Bde_4ZF  CKCKWDTCEMEFDTASE-LVAHVN-DHM-GSGKAS---YICQWRNCTRLQKPFTKRHKVQNHVRI-HTKERP
                      ZF1                                    ZF2

Afu_FG   FYCALPECDRSFTRSDALAKHMRTVHETEA
Nc__FG   FYCYLPECDRAFTRSDALAKHMRTVHETEA
Yl__FG   FYCSVPECDRNFTRSDALAKHMRTVHETEQ
Mgl_FG   FTCPRPECDKSFTRSDALAKHMRVQHNMPP
Um__FG   FTCPRPECDKSFTRSDALSKHMRVQHQIIT
Ro__FG   FVCPKP-----------------------
Ecu_FG   FECHL--CGKDYTRSDPLKKHLLR-HEAVD
Ca__FG   YFCPVPECEKHFTRSDALTKHVKAVHDLHS
Lbi_FG   FVCSVPECDKSFARPDSLHKHLRNQHNISP
Eb__FG   FKCQY--CFNLMATEESLKKHVLS-----
Alo_FG   FKCQK--CSKSYTRSDALNKHLKS-HKIAE
Cn__FG   YSCTIPGCNKAFARTDALNKHKRTVHADVT
Hs_G3    HKCTFEGCTKAYSRLENLKTHLRS-HTGEKPYVCEHEGCNKAFSNASDRAKHQNRTHS
Ro_4ZF   FVCTVIGCNKTFSRPDSLSTHIKT-HSDCRPYLCSMPGCNKAYYHSRSLRKHIKSTHM
Bde_4ZF  YICTMEDCGKTFSRLDGLNTHIRT-HSSVKPYICETPGCGKAYFHSRSLRKHERI-H
                     ZF3                         ZF4
```
