# Supplementary material for: Characterization of the tandem CWCH2 sequence motif: a hallmark of inter-zinc finger interactions
Source: BMC Evol Biol. 2010 Feb 19;10:53. doi: 10.1186/1471-2148-10-53 (PMC2837044; doi:10.1186/1471-2148-10-53)
Supplement: Additional file 8 — Sequence alignment of Twincl zinc finger domain. Based on its structural features, we named this gene family Twincl (Tandem CWCH2 protein C-terminal). Afu, Aspergillus fumigatus; Nf, Neosartorya fischeri; Cim, Coccidioides immitis; Pn, Phaeosphaeria nodorum; Gz, Gibberella zeae; Mgr, Magnaporthe grisea; Nc, Neurospora crassa. [file 1471-2148-10-53-S8.PDF]

```
Afu Twc YTCGWEKCQAELHNLEMLKKHISKIHVP-----------------YT
Nf  Twc YTCGWEKCQAELHNLEMLKKHIFKVHVP-----------------YT
Cim Twc YICSWKDCNAKLHNLETLKKHVVKLHVPP-------------SN-HS
Pn  Twc YKCKWQNCTADLHNLETLKKHVFKVHRKE-------------TLRNT
Gz  Twc FLCEWMDCKAELHNLETLRRHVYKVHGD------------------S
Mgr Twc FLCEWKDCKAELQNMDTLRRHVRKVHGR------------------E
Nc  Twc FLCEWEGCPAELHNFETLRKHVLVVHGDYRQPHQHHLLSAREQPQEP
                    ZF1

Afu Twc LTCQWKDCTFREN------------------LPAVQLYKHVLSEHVVSIAW
Nf  Twc LTCQWKDCTFREN------------------LPAVQLYKHVLSEHVVSIAW
Cim Twc TPCQWSGCASSRSQHL---------------FGAEELQEHLDKMHLRPMAW
Pn  Twc LECLWGDCGKEVTNYDPNTNMRIEKHTPHSFDLESNWRNHVQETHFDPLSW
Gz  Twc VECLWGKCGRLEEPPE--------------FEDDEGFNDHVEEAHLVPLSW
Mgr Twc EVCRWSTCASSGSTEVFG--------------TNDEFIGHVEHAHLVPFQW
Nc  Twc KTCKWASCHSKRLQSELP---------PLTLPTRSHFEAHVNESHLIPFLW
                          ZF2
```
