# Supplementary material for: Characterization of the tandem CWCH2 sequence motif: a hallmark of inter-zinc finger interactions
Source: BMC Evol Biol. 2010 Feb 19;10:53. doi: 10.1186/1471-2148-10-53 (PMC2837044; doi:10.1186/1471-2148-10-53)
Supplement: Additional file 9 — Sequence alignment of Clr1 zinc finger domain. Spo, Schizosaccharomyces pombe; Sj, Schizosaccharomyces japonicus; Cn, Cryptococcus neoformans. [file 1471-2148-10-53-S9.PDF]

```
Spo Clr1 YRCQWEGCLANLHSLENFIKHVLLLHHPKSCSV---------------------------------V
Sj  Clr1 YKCVWVGCEAVLHSFENLYNHCITAHCIEKEGA---------------------------------F
Cn  Clr1 TRCHWGKCEAILNSWAVLEKHIFQSHFHPNRTLPEMVVNGERK-----------------------I
Cn  Clr1 RECRWDDCNAVLGSENTLGMHV-HRHLANEEGAKDTWKMQLGMSSWIFTGYKGGRMKTEAVPNEYLY
                       ZF1

Spo Clr1 KCLWA--SCDMVLPS--EEFEMHLR-GHLNNIRLNCEVSNCKK-CFSNYEDMFKHLQHSHLPFKFTP
Sj  Clr1 PCFWQ--ACKASPSKSREQWKLHVH-VHLNSLVYSCPMRGCKE-TFNNYSELSSHFEAQHSHSEYEP
Cn  Clr1 RCLWGDGECQEAFGTRNE-LHQHVLVLHMKFVSARCPFGGCEYNGHDFNQFM-QHVNLIHS-T-ATP
Cn  Clr1 KCLWRK--CDEPCFTTKEKLLQHMLSKHV-SGKLICPYGWCRYACPSANTLA-RHITKIHAKTNDRP
                       ZF2                             ZF3
```
