# Supplementary material for: Characterization of the tandem CWCH2 sequence motif: a hallmark of inter-zinc finger interactions
Source: BMC Evol Biol. 2010 Feb 19;10:53. doi: 10.1186/1471-2148-10-53 (PMC2837044; doi:10.1186/1471-2148-10-53)
Supplement: Additional file 10 — Sequence alignment of Monosiga, Dictyostelium, and plant gene. (A) Zinc finger domain alignment of Dictyostelium discoideum hypothetical protein (DdHp) and Monosiga brevicollis tCWCH2. DdHp2 contains two tCWCH2 domains {ZF12 (ZF1-ZF2) and ZF34 (ZF3-ZF4)}. (B) Plant REF6 Physcomitrella patens (XP_001771543) gene has tCWCH2 sequence. It has strong homology to Arabidopsis REF6 (NP_680116), Oryza sativa gene (NP_001045137) and Vitis vinifera (CAO64178) gene, but the tryptophan residue is not conserved in Arabidopsis and other plants. An alignment of zinc finger domain of these genes is shown. Amino acid identity between sequences is shown by a dot (.). [file 1471-2148-10-53-S10.PDF]

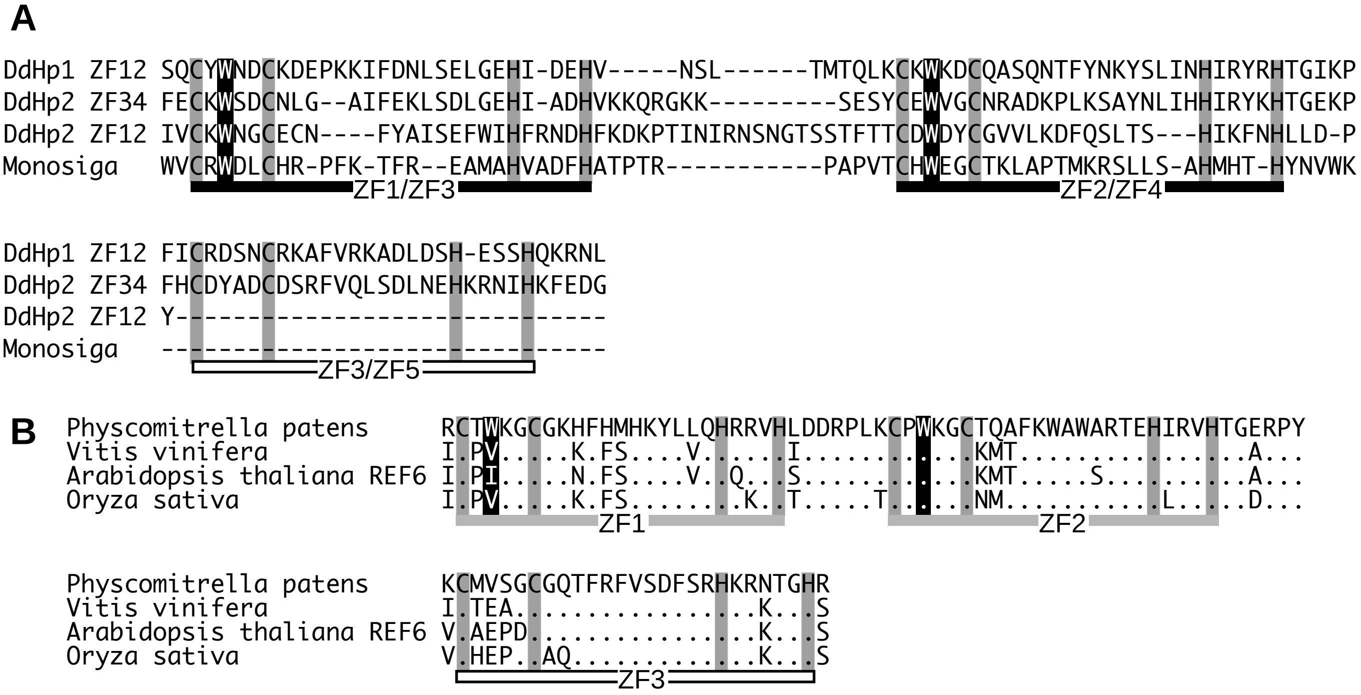
A
DdHp1 ZF12 SQCYWNDCKDEPKKIFDNLSELGEHI-DEHV-----NSL------TMTQLKCKWKDCQASQNTFYNKYSLINHIRYRHTGIKP
DdHp2 ZF34 FECKWSDCNLG--AIFEKLSDLGEHI-ADHVKKQRGKK---------SESYCEWVGCNRADKPLKSAYNLIHHIRYKHTGEKP
DdHp2 ZF12 IVCKWNGCECN----FYAISEFWIHFRNDHFKDKPTINIRNSNGTSSTFTTCDWDYCGVVLKDFQSLTS---HIKFNHLLD-P
Monosiga WVCRWDLCHR-PFK-TFR--EAMAHVADFHATPTR-----------PAPVTCHWEGCTKLAPTMKRSLLS-AHMHT-HYNVWK
ZF1/ZF3
ZF2/ZF4
DdHp1 ZF12 FICRDSNCRKAFVRKADLDSH-ESSHQKRNL
DdHp2 ZF34 FHCDYADCDSRFVQLSDLNEHKRNIHKFEDG
DdHp2 ZF12 Y------------------------------
Monosiga -------------------------------
ZF3/ZF5
B
Physcomitrella patens RCTWKGCGKHFHMHKYLLQHRRVHLDDRPLKCPWKGCTQAFKWAWARTEHIRVHTGERPY
Vitis vinifera I.PV.....K.FS....V......I...........KMT...............A...
Arabidopsis thaliana REF6 I.PI.....N.FS....V..Q...S...........KMT.....S.........A...
Oryza sativa I.PV.....K.FS.......K..T.....T.....NM...........L.....D...
ZF1
ZF2
Physcomitrella patens KCMVSGCGQTFRFVSDFSRHKRNTGHR
Vitis vinifera I.TEA...............K...S
Arabidopsis thaliana REF6 V.AEPD..............K...S
Oryza sativa V.HEP..AQ...........K...S
ZF3
