# Supplementary material for: Characterization of the tandem CWCH2 sequence motif: a hallmark of inter-zinc finger interactions
Source: BMC Evol Biol. 2010 Feb 19;10:53. doi: 10.1186/1471-2148-10-53 (PMC2837044; doi:10.1186/1471-2148-10-53)
Supplement: Additional file 11 — Sequence alignment for phylogenic tree analysis. Conserved cysteine and histidine residues are indicated with an asterisk (*). Afr, Artemia franciscana; Afu, Aspergillus fumigatus; Aga, Anopheles gambiae; Alo, Antonospora locustae; Ape, Asterina pectinifera; Bde, Batrachochytrium dendrobatidis; Bfl, Branchiostoma floridae; Cin, Ciona intestinalis; Cn, Cryptococcus neoformans; Co, Corbicula sp.; Da, Dicyema acuticephalum; Dd, Dictyostelium discoideum; Dj, Dugesia japonica; Dm, Drosophila melanogaster; Hr, Halocynthia roretzi; Hs, Homo sapiens; Hv, Hydra vulgaris; Lbl, Loligo bleekeri; Mms, Mus musculus; Nf, Neosartorya fischeri; Nve, Nematostella vectensis; Oo, Octopus ocellatus; Pi, Pandinus imperator; Ro, Rhizopus oryzae; Sc, Saccharomyces cerevisiae; Sm, Schistosoma mansoni; Sp, Strongylocentrotus purpuratus; Sso, Spisula solidissima; Ssu, Scolionema suvaense; Ta, Trichoplax adhaerens; Tt, Tubifex tubifex; Um, Ustilago maydis; Xl, Xenopus laevis; Yl, Yarrowia lipolytica. [file 1471-2148-10-53-S11.PDF]

```
  Afu_ZafA   CKWTTTSHG--------------------VKRSCG----ATFADACALQEHLVANHMGTVEGAKGTGYYCCWEGCHR--PDEPFSQKSKLQGHFL-THS-NYKNFKC--SVCGKLFARQATLERHERS-H
   Nf_ZafA   CKWTATSHG--------------------VKRSCG----ATFADPCALQEHLVANHMGTVEGAKGTGYYCCWEGCHR--PDEPFSQKSKLQGHFL-THS-NYKNFKC--SVCGKLFARQATLERHERS-H
   Hs_Aebp   CCWDQ----------------------------CQ----ACFNSSPDLADHIRSIHVDGQ--RGGV-FVCLWKGCKV--YNTPSTSQSWLQRHML-THS-GDKPFKCVVGGCNASFASQGGLARHVPT-H
  Mms_Aebp   CCWDQ----------------------------CQ----ACFNSSPDLADHIRSIHVDGQ--RGGV-FVCLWKGCKV--YNTPSTSQSWLQRHML-THS-GDKPFKCVVGGCNASFASQGGLARHVPT-H
   Xl_Aebp   CCWDH----------------------------CQ----TPFSCSPDLADHIRSIHVDGQ--HGGV-YVCYWKGCKV--YNTPSTSHSWLQRHML-THS-GDKPFKCVVGDCNASFASQGGLARHVPT-H
  Bfl_Aebp   CKWEQ----------------------------CD----QSVASSSDLADHLSNVHVRE---EGEK-VVCLWQGCRV--YNMPARSRSWLSRHVL-QHS-GDRPFKCMIEGCRAAFASQGGLARHVPT-H
   Sp_Aebp   CRWES----------------------------CG----EETDTSADLADHVRAEHAQKQIKKGGKKFVCLWDGCKV--YNTPSMSATWLPKHVL-THC-GDKPFRCVFAGCNQSYRTEKGLMRHCQS-H
  Cin_Aebp   CAWDA----------------------------CS----SVFITSRDLHDHICSVHVDTTTKR----FCCLWSGCKV--YNTPARSVEWLKQHVG-RHT-GTKRFRCLLDGCDASFNSQNGLARHVPS-H
   Ta_Aebp   CRWFG----------------------------CN----NSFESTEELRDHLQKIHIDTHDVEAK--YACLWQGCKV--YNKPSVSRNWLVQHVC-KHT-GDRLIKCLIDGCNMTFATQNGLARHVPS-H
   Dm_Aebp   CYWDK----------------------------CN----KKHESNSKLLDHMQTHHVNTQ-TGP---FACLWVGCKV--YNKESCSRRWLERHVL-SHG-GSKQFKCIVEGCGLRFGSQLALQKHVNN-H
  Nve_Aebp   CKWNE----------------------------CT----AHISCTSDLSEHVHQSHVEPMVTQDV--YVCLWQGCKV--FNKPSCSHSWLSKHMN-SHT-GDKPWKCVIGGCSLSFASCEGLSRHVSQ-H
    Ta_Zic   CRWINQDRQ-----------------SSRFGKVCN----RTFGSMQEIVSHLNVDHVGA-PDQTT--HTCHWENCQR--AGKPFKAKYKLVNHIR-VHT-GEKPFQCPFPSCCKLFARSENLKIHKRT-H
  Nve_ZicA   CKWIDCTEK--------------------VSLLCD----KVFSCMDELVKHITIEHVNG-RDSTQ--HVCCWESCDR--AGKPFKAKYKLVNHIR-VHT-GEKPFVCPFSSCNKLFARSENLKIHKRT-H
   Dj_ZicA   CLWIDPNQR-------------------DGSKPCL----KLYSSILDIVTHLTMDHVGG-PEQLD--HTCYWKDCPR--DCKAFKAKYKLVNHIR-VHT-GEKPFQCPFSNCGKLFARSENLKIHKRT-H
   Dj_ZicB   CLWLDKHMK-------------------EERRTCG----KLFYTINEIVNHLTLDHVGG-PEQLD--HTCYWKNCTR--DFKPFKAKYKLVNHLR-VHT-GEKPFQCPFLSCGKLFARSENLKIHKRT-H
    Sm_Zic   CQWIDPVPT----------------IPGSIPKPCS----RVFDSVTEIVNHITLEHVGG-PEQLD--HTCYWKDCIR--DGKPFKAKYKLVNHIR-VHT-GEKPFPCPFSGCMKVFARSENLKIHKRT-H
    Pi_Zic   CLWIDQEAP-------------------NPKKTCS----KTFNSMHEIVTHITVEHVGG-PECTN--HACYWQDCVR--NGRPFKAKYKLVNHIR-VHT-GEKPFPCPFPNCGKVFARSENLKIHKRT-H
   Afr_Zic   CLWVDPEQP-------------------SPKKTCG----KTFGSMHEIVTHITVEHVGG-PECTN--HACFWQGCVR--NGRAFKAKYKLVNHIR-VHT-GEKPFPCPFPGCGKVFARSENLKIHKRT-H
    Dm_Zic   CLWIDPDQPGLVPP--------------GGRKTCN----KVFHSMHEIVTHLTVEHVGG-PECTT--HACFWVGCSR--NGRPFKAKYKLVNHIR-VHT-GEKPFACPHPGCGKVFARSENLKIHKRT-H
   Ape_Zic   CLWIEQEQP-------------------EPKKPCN----KTFTTMHEIVTHITVEHVGG-PEQTN--HTCFWQNCSR--DQKPFKAKYKLVNHIR-VHT-GEKPFPCPFPGCGKVFARSENLKIHKRT-H
    Sp_Zic   CLWIDQDLP-------------------EPRKPCN----KTFTTMHEIVTHITVEHVGG-PEQTN--HTCFWQNCSR--EQKPFKAKYKLVNHIR-VHT-GEKPFPCPFPGCGKVFARSENLKIHKRT-H
    Oo_Zic   CMWVETDQP-------------------EPKKPCN----KTFPSMHEIVAHITVEHVGG-PEQTN--HSCYWQNCTR--DGRPFKAKYKLVNHIR-VHT-GEKPFPCPFPGCGKVFARSENLKIHKRT-H
   Lbl_Zic   CLWVDTDQP-------------------EPKKSCN----KTFPSMHEIVAHITVEHVGG-PEQTN--HSCYWQNCTR--DGRPFKAKYKLVNHIR-VHT-GEKPFPCPFPGCGKVFARSENLKIHKRT-H
   Sso_Zic   CLWLDKDQP-------------------EPKKPCN----KTFTTMHEIVTHITVEHVGG-PEQTD--HACYWQNCPR--DGRPFKAKYKLVNHIR-VHT-GEKPFPCPFPGCGKVFARSENLKIHKRT-H
    Co_Zic   CLWLDKDQP-------------------EPKKPCN----KTFTTMHEIVTHITVEHVGG-PEQTD--HACYWQSCPR--DGRPFKAKYKLVNHIR-VHT-GEKPFPCPFPGCGKVFARSENLKIHKRT-H
   Bfl_Zic   CLWIDPDQP-------------------EPKKPCN----KSFSTMHEIVTHVTVEHVGG-PECTN--HACFWKDCPR--DGRAFKAKYKLVNHIR-VHT-GEKPFPCPFPGCGKLFARSENLKIHKRT-H
    Tt_Zic   CMWIDVEPA--VPPHYGPRGLFISDDSRRVEKPCG----KIYSSMHEIVTHITVDHVGG-PEQTN--HACLWRGCSR--QLKPFKAKYKLVNHIR-VHT-GEKPFPCPFPGCGKVFARSENLKIHKRT-H
  Mms_Zic3   CKWLDPEELAG-----------PPASADSGVKPCS----KTFGTMHELVNHVTVEHVGG-PEQSS--HVCFWEDCPR--EGKPFKAKYKLINHIR-VHT-GEKPFPCPFPGCGKVFARSENLKIHKRT-H
   Hs_Zic3   CKWIDPDELAGLPPPPPPPPPPPPPPPAGGAKPCS----KTFGTMHELVNHVTVEHVGG-PEQSS--HVCFWEDCPR--EGKPFKAKYKLINHIR-VHT-GEKPFPCPFPGCGKVFARSENLKIHKRT-H
   Hs_Zic2   CKWIDPEQLS------------------NPKKSCN----KTFSTMHELVTHVSVEHVGG-PEQSN--HVCFWEECPR--EGKPFKAKYKLVNHIR-VHT-GEKPFPCPFPGCGKVFARSENLKIHKRT-H
  Mms_Zic2   CKWIDPEQLS------------------NPKKSCN----KTFSTMHELVTHVSVEHVGG-PEQSN--HVCFWEECPR--EGKPFKAKYKLVNHIR-VHT-GEKPFPCPFPGCGKVFARSENLKIHKRT-H
   Xl_Zic2   CKWIDPEQLN------------------NPKKSCN----KTFSTMHELVTHMSVEHVGG-PEQSN--HICFWEECAR--EGKPFKAKYKLVNHIR-VHT-GEKPFPCPFPGCGKVFARSENLKIHKRT-H
   Xl_Zic3   CKWIDQDQ--------------------SSKKPCS----KTFSTMHELVNHVAVEHVGG-PEQSN--HICFWEECAR--EGKPFKAKYKLVNHIR-VHT-GEKPFPCPFPGCGKVFARSENLKIHKRT-H
  Nve_ZicE   CEWVDPQNY-------------------GKGKICG----KQFSVLHDIVRHINDEHVS---QNDSPLHVCHWRNCTR--NGLPFKAKYKLVNHIR-VHT-GEKPFPCPFPGCGKLFARSENLKIHKRT-H
   Hv_Zic1   CMWIEHHGF-------------------SKMKPCG----RQFSNMLDIVNHLSEEHVQ-TADTGNGLYVCQWQNCPR--NGLPFKAKYKLVNHLR-VHT-GEKPFPCPFPGCGKLFARSENLKIHKRT-H
   Ssu_Zic   CMWID--ST-------------------TKGKPCG----KQFFVMMDIVQHLAEDHVGI-NESTE--HICYWKDCPR--SGMAFKAKYKLINHLR-VHT-GEKPFPCPFPGCGKLFARSENLKIHKRT-H
   Da_ZicA   CKWQTEHDE-----------------NIYSKNICG----KIFISLNDFVHHITADHVGG-SDRTE--HCCMWLDCTR--TCKPFKAKYKLVNHIR-VHT-GEKPFVC--NSCGKFFARSENLKIHQRT-H
   Da_ZicB   CKWQIENDK-----------------NIYSKNICE----KFFISLNDFVHHITADHVGG-SDRKE--HCCMWLDCTR--TCKPFKAKYKLVNHIR-VHT-GEKPFVC--NCCGKFFARSENLKIHQRT-H
    Hr_Zic   CKWIVSGKS-----------------GEENAITCD----REFYSMNQLVDHVTVDHVGG-HDQAD--HTCYWKDCTR--E-KSFQAKYKLVNHIR-VHT-GEKPFICLFPNCGKVFARSENLKIHKRT-H
   Cin_Zic   CKWRNMKNG---------------------GRSCD----VIFHDMHELVNHVTRDHVGG-MDQTD--HTCYWKDCSR--KRKGFKAKYKLVNHIR-VHT-GEKPFVCPFPDCGKTFGRSENLKIHQRT-H
   Cin_Zic   CKWTKPLTN-LYENADACQQYPKVDNQHINGEPCN----LIFHSMLDLVTHVGRDHVGG-PEHTD--HACYWQDCAR--ECKPFKAKYKLINHIR-VHT-GEKPFQCPYMGCGKVFARSENLKIHKRT-H
    Hr_Zic   CKWMYPANK-----QQLGKSTVPNEAPEVTEEPCN----RVFVTLLDLVTHISLNHIGG-SEQID--HTCYWQNCSR--GKKSFKAKYKLVNHTR-VHT-GERPFSCPFPGCGKMFARSENLKIHKRI-H
 Mms_Glis3   CRWID----------------------------CS----ALYDQQEELVRHIEKVHIDQ-RKGED--FTCFWTGCPR--RYKPFNARYKLLIHMR-VHS-GEKPNKCTFEGCKKAFSRLENLKIHLRS-H
  Hs_Glis3   CRWID----------------------------CS----ALYDQQEELVRHIEKVHIDQ-RKGED--FTCFWAGCPR--RYKPFNARYKLLIHMR-VHS-GEKPNKCTFEGCEKAFSRLENLKIHLRS-H
  Sp_Glis3   CRWID----------------------------CN----AFFEEQEELVRHIEKVHIDQ-RKGED--FTCFWQGCTR--RFKPFNARYKLLIHMR-VHS-GEKPNKCTFEGCNKAFSRLENLKIHLRS-H
 Cin_Glis3   CMWAE----------------------------CN----LFFQDQEDLVKHIEKVHIDQ-RKGEE--FTCYWMNCPR--QYKPFNARYKLLIHMR-VHS-GERPNKCTFEGCNKAFSRLENLKIHLRS-H
 Nve_Glis3   CRWLG----------------------------CD----AVYSEQDDLVRHIEKVHIDQ-RKADD-LYICYWQDCSR--QTKPFNARYKLVIHMR-VHS-GEKPNKCTHPGCNKAFSRLENLKIHMRS-H
 Aga_Glis3   CLWSG----------------------------CN----MEFADQQQLVSHIEKQHVEP-KRGE--VFGCQWLECPR--QHRPFNARYKLLIHMR-VHS-GEKPNKCPFPTCKKAFSRLENLKIHQRS-H
  Dm_Glis3   CRWTG----------------------------CD----EEFPHQQAFVEHIEKCHVDV-RKGED--FSCFWLDCPR--RYKPFNARYKLLIHMR-VHS-GEKPNKCPFPGCNKAFSRLENLKIHQRS-H
    Dm_Gli   CHWRS----------------------------CR----IEFITQDELVKHINNDHIQTNKKA----FVCRWEDCTR--GEKPFKAQYMLVVHMR-RHT-GEKPHKCTFEGCFKAYSRLENLKTHLRS-H
   Aga_Gli   CHWRE----------------------------CS----LEFNTQDELVKHINNDHIHANKKS----FVCRWEDCSR--DEKPFKAQYMLVVHMR-RHT-GEKPHKCTFEGCCKAYSRLENLKTHLRS-H
  Mms_Gli3   CHWEG----------------------------CT----REFDTQDQLVHHINNDHIHGEKKE----FVCRWLDCSR--EQKPFKAQYMLVVHMR-RHT-GEKPHKCTFEGCTKAYSRLENLKTHLRS-H
   Hs_Gli3   CHWEG----------------------------CA----REFDTQEQLVHHINNDHIHGEKKE----FVCRWLDCSR--EQKPFKAQYMLVVHMR-RHT-GEKPHKCTFEGCTKAYSRLENLKTHLRS-H
   Xl_Gli3   CHWEG----------------------------CS----REFDTQEQLVHHINNDHIHGEKKE----FVCRWLDCSR--EQKPFKAQYMLVVHMR-RHT-GEKPHKCTFEGCSKAYSRLENLKTHLRS-H
  Bfl_Gli2   CHWDG----------------------------CS----KEFDTQDQLVHHINNDHIHGEKKE----FVCRWSECTR--EQKPFKAQYMLVVHMR-RHT-GEKPHKCTFEGCNKAYSRLENLKTHLRS-H
   Xl_Gli1   CHWES----------------------------CT----KEFDTQEHLVHHINNEHIHGEKKE----FVCHWQDCSR--ELRPFKAQYMLVVHMR-RHT-GEKPHKCTFEGCNKAYSRLENLKTHLRS-H
  Cin_Gli3   CFWEN----------------------------CS----LEFDTQEQLVHHINNDHIHGDKKE----FVCRWRGCAR--DQRPFKAQYMLVVHMR-RHT-GEKPHRCTFDGCTKAYSRLENLKTHLRS-H
  Mms_Gli1   CRWDG----------------------------CS----QEFDSQEQLVHHINSEHIHGERKE----SVCHWGGCSR--ELRPFKAQYMLVVHMR-RHT-GEKPHKCTFEGCRKSYSRLENLKTHLRS-H
   Hs_Gli1   CRWDG----------------------------CS----QEFDSQEQLVHHINSEHIHGERKE----FVCHWGGCSR--ELRPFKAQYMLVVHMR-RHT-GEKPHKCTFEGCRKSYSRLENLKTHLRS-H
   Nve_Gli   CHWKD----------------------------CT----IVFDSQDSLVKHVNGDHIKKEKRD----FTCYWQDCSR--EQRPFKAQYMLVVHMR-RHT-GEKPHKCYHEGCTKAYSRLENLKTHLRS-H
  Hs_Glis2   CRWAK----------------------------CN----QLFELLQDLVDHVNDYHVKPEKDAG---YCCHWEGCAR--HGRGFNARYKMLIHIR-THT-NEKPHRC--PTCSKSFSRLENLKIHNRS-H
 Mms_Glis2   CRWAK----------------------------CN----QLFELLQDLVDHVNDHHVKPEQDAR---YCCHWEDCAR--HGRGFNARYKMLIHIR-THT-NEKPHRC--PTCNKSFSRLENLKIHNRS-H
 Nve_Glis2   CKWES----------------------------CC----HHFLTLDDLVNHVNEYHITFDKDSE---YRCRWQGCNR--NGKGFNARYKMLIHMR-THT-GERPHKCTFDSCEKSFSRLENLKIHQRS-H
 Aga_Glis2   CRWEN----------------------------CY----CVFFKLEDLASHVTQKHAVIGL-DGL--YYCRWERCLR--QDRGFNARYKMLVHVR-THT-KEKPHQC--GKCGKCFSRAENLKIHLRS-H
   Dm_Glis   CNWTD----------------------------CD----RVFDTLDALAQHVTQRHAIASLTDGL--YYCRWRGCQR--SERGFNARYKMLVHTR-THT-KEKPHRC--HLCEKSFSRAENLKIHIRS-H
 Afu_Fungl   CRWEG----------------------------CNV--GDLGN-MDDLVQHIHNDHVGNRQKR----YSCEWSDCPR--KGQTHASGYALRAHMR-SHT-REKPFYCALPECDRSFTRSDALAKHMRTVH
  Nf_Fungl   CRWEG----------------------------CNV--GDLGN-MDDLVQHIHNDHVGNRQKR----YSCEWSDCPR--KGQTHASGYALRAHMR-SHT-REKPFYCALPECDRSFTRSDALAKHMRTVH
  Um_Fungl   CQWND----------------------------CG---ETFNS-LQPFIDHLHNEHIGIHKSK----YMCEWTGCIR--KGKPQTSRFALLSHLR-SHT-GEKPFTCPRPECDKSFTRSDALSKHMRVQH
  Cn_Fungl   CQWGE----------------------------CQ---GDFDS-KQEFYGHVK-DHI-NASKE----YACEWRTCSR--VGHKQGRSL-LLTHIR-GHT-GERPYSCTIPGCNKAFARTDALNKHKRTVH
   Cn_PacC   CKWTG----------------------------CS----HISDSPDELYDHLCTVHVGRKSTNNLC-LTCGWENCGT-----KCVKRDHITSHLR-VHT-PLKPHPCAV--CGKTFKRPQDLKKHERI-H
   Um_PacC   CRWDD----------------------------CG----KIFYDPEVVYKHLCDDHVGRKSTNNLC-LTCKWEGCDV-----SCAKRDHITSHIR-VHT-PLKPHNCDA--CGKTFKRPQDLKKHERI-H
   Sc_PacC   CKWDN----------------------------CG----MIFNQPELLYNHLCHDHVGRKSHKNLQ-LNCHWGDCTT----KTE-KRDHITSHLR-VHV-PLKPFGCST--CSKKFKRPQDLKKHLKI-H
   Ro_PacC   CKWSN----------------------------CT----LLFDDPEQLYLHLTNDHVGRKSTGNLC-LTCHWENCDV-----TVIKRDHITSHLR-VHV-PLKPHKCQV--CNKSFKRPQDLKKHEKI-H
   Yl_PacC   CKWGP----------------------------CG----KTFGSAEKLYAHLCDAHVGRKCTHNLS-LVCNWDNCGI-----VTVKRDHITSHIR-VHV-PLKPYKCDF--CTKSFKRPQDLKKHVKT-H
  Afu_PacC   CLWQG----------------------------CS----EKCPSAEALYDHICERHVGRKSTNNLN-LTCQWGSCRT-----TTVKRDHITSHIR-VHV-PLKPHKCEF--CGKAFKRPQDLKKHVKT-H
  Bde_PacC   CLWVFPSR-------------------------CV----YSFASSDLLYRHICDDHIGRKTTGNLC-LDCHWDRCTV--FR---SKRDHLTSHIR-VHL-KLRPHACTI--CGRTFKRMQDLNKHSRI-H
    Sc_Zap   CQWDG----------------------------CN----KSFSSAQELNDHLEAVHL-TRGKSE---YQCLWHDCHR-----TFPQRQKLIRHLK-VHS-KYKPYKCKT--CKRCFSSEETLVQHTRT-H
   Yl_ZafA   CQWVLEKDP---------------------KHVCK----LQFSSAKDLSDHVIEKHIGSR-KPE---YSCSWDGCER--CDRPFTQRQKVVRHLQ-THT-KHRPHQCPV--CHYRFAEESVLKQHMRI-H
   Ro_ZafA   CRWQN----------------------------CS----QRFEGFTKLTNHVSKDHVGSG---KSE-YQCYWTSCDR--RGRGFTQRQKIMRHIQ-THT-GAKPYQCQV--CQKRFSESNVMIQHMRT-H
   Um_ZafA   CRWMG----------------------------CS----ASFDSHSALTDHIETEHVGSG---QAQ-YECKWIGCARYVSGQKFSQKQKVLRHIQ-THT-GDRPFKC-SE-CGKRFSEQNTLAQHMRT-H
Mms_TFIIIA   CSFPD----------------------------CS----ASYNKAWKLDAHLC-KHTGE----RP--FVCDYEGCGK-----AFIRDYHLSRHVL-IHT-GEKPFVCADDGCNQKFNTKSNLKKHIERKH
 Hs_TFIIIA   CSFPD----------------------------CS----ANYSKAWKLDAHLC-KHTGE----RP--FVCDYEGCGK-----AFIRDYHLSRHIL-THT-GEKPFVCAANGCDQKFNTKSNLKKHFERKH
 Xl_TFIIIA   CSFAD----------------------------CG----AAYNKNWKLQAHLC-KHTGE----KP--FPCKEEGCEK-----GFTSLHHLTRHSL-THT-GEKNFTCDSDGCDLRFTTKANMKKHFNRFH
 Dd_TFIIIA   CSY------------------------------CG----KVFPRASKLEYHIRT-HTGE----KP--YHCKFEGCGK-----SFSRSHHLSYHEK-THSPPQEKIPCPIVGCTNTFVFKHHLQTHIKSKH
Afu_TFIIIA   CPFDG----------------------------CT----KAFNRPARLQEHLRS-HNNE----RI--FKCTFEECDK-----TFLRASHLNHHIKSAHT-GVRDYVCDRPGCGKSFVTGSRLRRHLAA-H
 Nf_TFIIIA   CPFDG----------------------------CT----KAFNRPARLQEHLRS-HNNE----RI--FKCTFEECDK-----TFLRASHLNHHIKSAHT-GVRDYVCDRPGCGKSFVTGSRLRRHLAA-H
 Yl_TFIIIA   CPEEG----------------------------CD----KAYSRPSLLKQHLRS-HYNE----RC--FVCTYEGCGK-----GFFRRSHLKAHTN-SHT-VAKRYHCSF--CAKGFNTRQHLLRHEVT-H
 Sc_TFIIIA   CDYDG----------------------------CD----KAFTRPSILTEHQLSVHQGL----R--AFQC--DKC-----AKSFVKKSHLERHLY-THS-DTKPFQCSY--CGKGVTTRQQLKRHEVT-H
 Sp_TFIIIA   CTHEG----------------------------CT----KAFTRQPHLQRHL-SSHSSQ----N--DLLCEEVGC-----GAAFSNKDNLKKHIKRVHQ--GRQYKCDYEGCGKTFHKHQHLTVHQYI-H
             *                                *                *    *                *    *                   *    *         *    *            *    *
```
