# Supplementary figures and images for: Characterization of the tandem CWCH2 sequence motif: a hallmark of inter-zinc finger interactions
Source: BMC Evol Biol. 2010 Feb 19;10:53. doi: 10.1186/1471-2148-10-53 (PMC2837044; doi:10.1186/1471-2148-10-53)

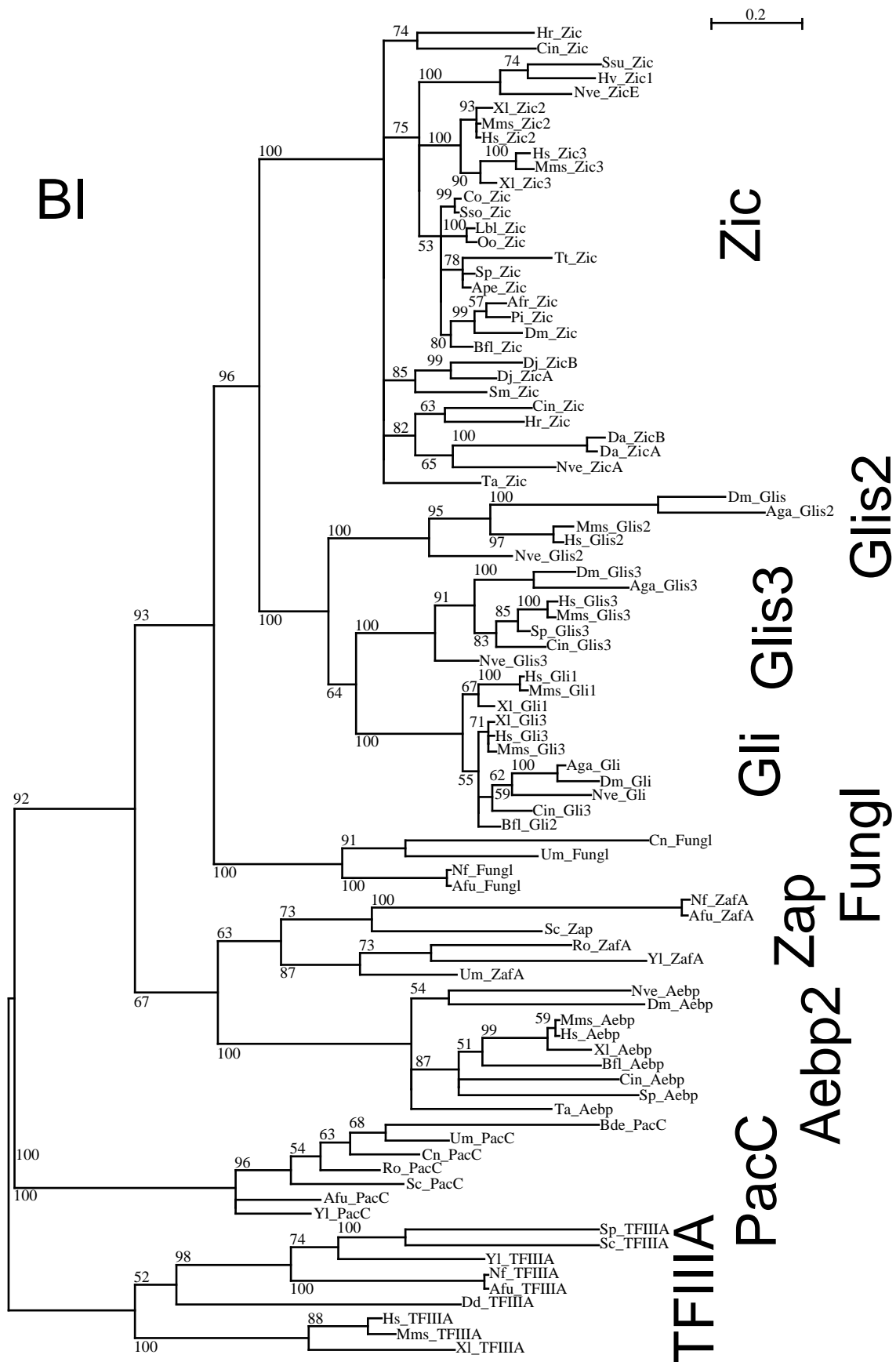

Supplement: Additional file 12 — BI tree of tCWCH2+1ZF. BI tree analysis was performed on 8 × 10^6^ generations using WAG model. Average standard deviation of split frequencies was lower than 0.001 at the end of the analysis. Abbreviations are the same as those in Additional file 11. [file 1471-2148-10-53-S12.PDF]

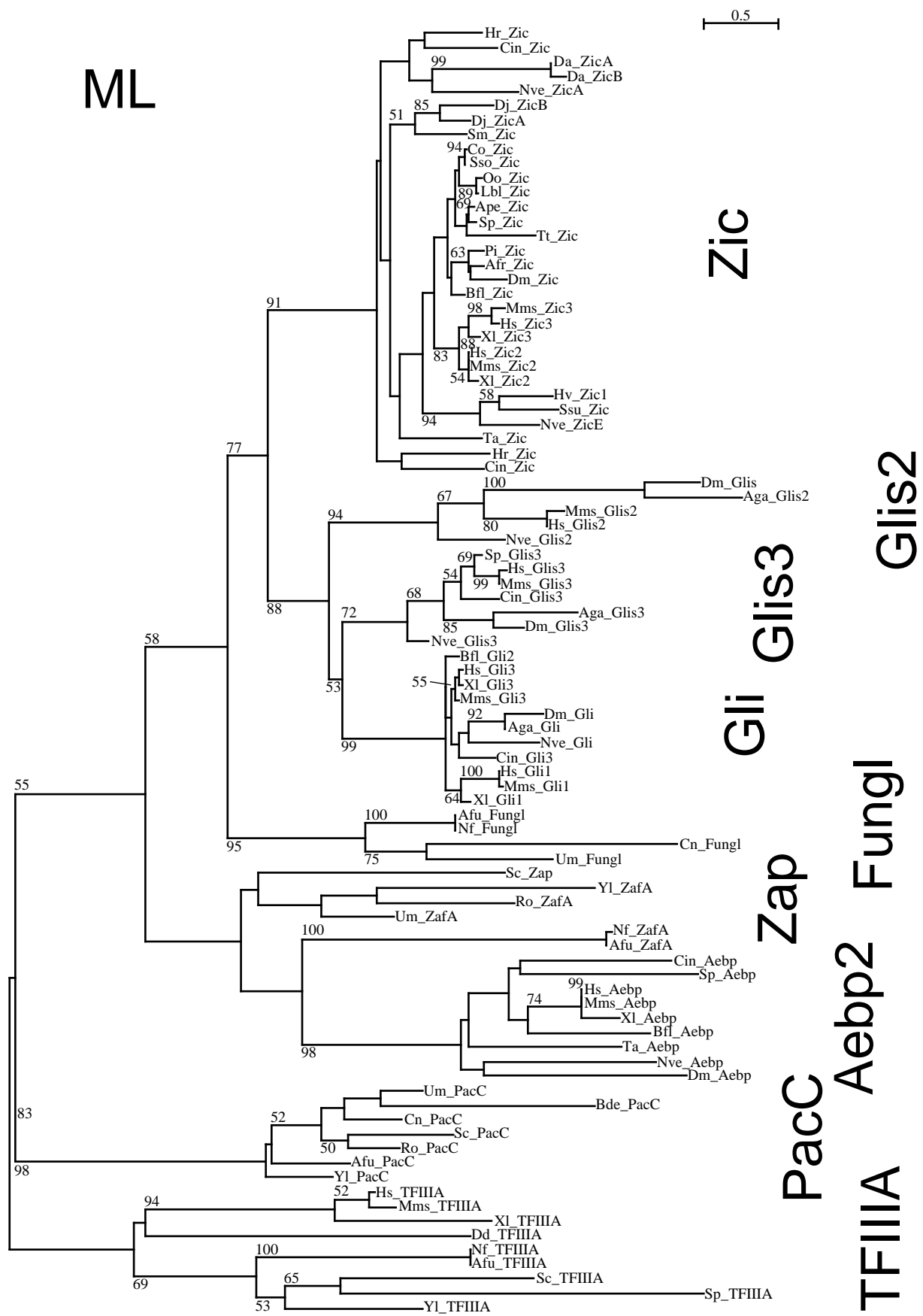

Supplement: Additional file 13 — ML tree of tCWCH2+1ZF. ML tree analysis was performed using WAG model with "empirical base frequencies", "maximum likelihood search", and "estimate proportion of invariable sites" options of RAxML [75]. 100 replicates were set for the bootstrap analysis. Abbreviations are the same as those in Additional file 11. [file 1471-2148-10-53-S13.PDF]

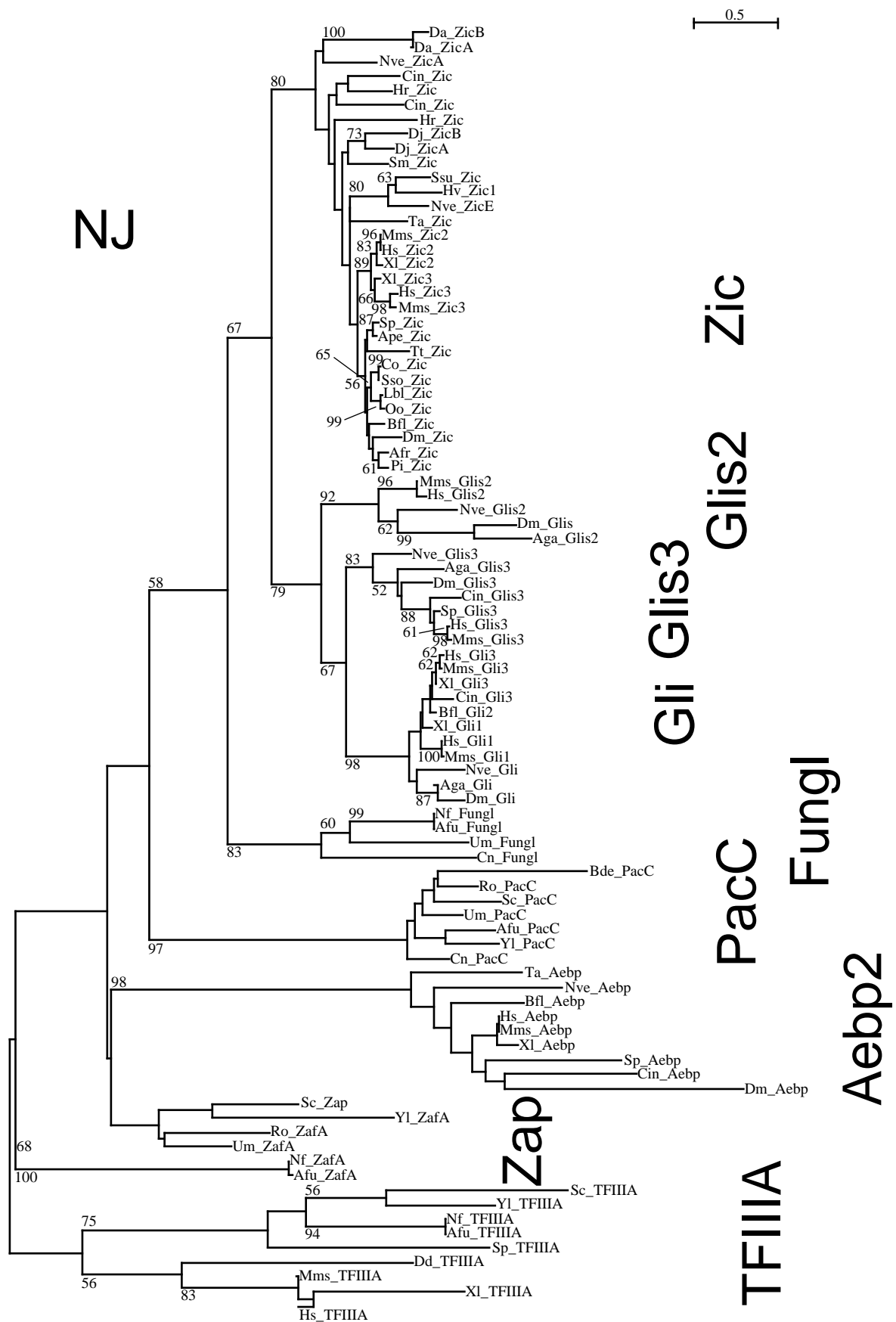

Supplement: Additional file 14 — NJ tree of tCWCH2+1ZF. NJ tree analysis was performed using JTT model and "pair wise deletion", "Rate among site = different (gamma distribution = 0.62)" options of MEGA4 (http://www.megasoftware.net/, [73,74]). The gamma distributions alpha parameter was calculated by "Tree-Puzzle" (http://www.tree-puzzle.de/, [72]). 1000 replicates were set for the bootstrap analysis. Abbreviations are the same as those in Additional file 11. [file 1471-2148-10-53-S14.PDF]
